# The role of ANGPTL4 in cancer: A meta-analysis of observational studies and multi-omics investigation

**DOI:** 10.1371/journal.pone.0320343

**Published:** 2025-04-15

**Authors:** Osama M. Younis, Abdalrahman S. Dhaydel, Wasfi F. Alghwyeen, Noor R. Abu Hantash, Leen M. Allan, Issam M. Qasem, Anwaar Saeed

**Affiliations:** 1 Division of Hematology & Oncology, Department of Medicine, University of Pittsburgh Medical Center (UPMC), Pittsburgh, Pennsylvania, United States of America; 2 School of Medicine, The Hashemite University, Al Zarqaa, Jordan; 3 School of Medicine, The University of Jordan, Amman, Jordan; 4 School of Medicine, Yarmouk University, Irbid, Jordan.; International University of Health and Welfare, School of Medicine, Japan

## Abstract

**Background:**

Angiopoietin-like protein 4 (ANGPTL4) plays a crucial role in processes such as angiogenesis, inflammation, and metabolism. Despite numerous studies suggesting its involvement in cancer, a definitive role remains unclear. We introduce the first comprehensive meta-analysis and pan-cancer bioinformatics study on ANGPTL4, aiming to unravel its implications across various cancer types.

**Methods:**

Moderate-to high-quality observational studies were retrieved from PubMed, Scopus, and Embase. A meta-analysis was conducted using the R package “meta.” Survival analysis was performed using GEPIA2 and TIMER2.0. Immune infiltration, mutational burden, and drug resistance analyses was done via GSCAlite. Co-expression and gene set enrichment analyses (GSEA) were carried out using cBioportal and enrichr, respectively.

**Results:**

Increased ANGPTL4 expression was linked to worse tumor grade (OR =  1.51, P = 0.023), stage (OR =  2.42, P < 0.001), lymph node metastasis (OR =  1.76, P = 0.012), vascular invasion (OR =  2.16, P = 0.01), and lymphatic invasion (OR =  2.20, P < 0.001). Furthermore, ANGPTL4 expression was linked to worse OS (HR = 1.40, 95% CI: 1.29,1.50, P = 0.0001). Single gene level analysis revealed that ANGPTL4 upregulated epithelial-to-mesenchymal transition (EMT) in 23 different cancers. Immune infiltration varied between cancer types, but increased infiltration of cancer-associated fibroblasts was observed in most cancers. Mutation analysis revealed increased alterations in TP53 and CDKN2A in cohorts with ANGPTL4 alterations. GSEA of co-expressed genes revealed involvement in hypoxia, EMT, VEGF-A complex, TGF-B pathways, and extracellular matrix organization.

**Conclusions:**

ANGPTL4 plays a significant role in tumor progression via its positive regulation of EMT and angiogenesis, while possibly harboring a TGF-B dependent role in systemic metastasis. Therefore, ANGPTL4 is a suitable target for future drug development.

## 1. Introduction

Cancer is a multifaceted disease that is growing in prevalence [1], with its multifaceted nature resulting in its difficulty to treat. Cancer development, also known as tumorigenesis, is attributed to normal cells acquiring mutations that provide them with limitless proliferative capabilities. This is further coupled with mutations that lead to metastasis, evasion of cellular death mechanisms, immune modulation, inflammatory control, and angiogenic properties that eventually result in full autonomy of the malignant entity called cancer [2,3].

Angiopoietin like 4 (ANGPTL4) is one of eight proteins that belong to the angiopoietin-like family (ANGPTL) [4]. The ANGPTL family is named after their shared amino acid sequence domains and cleavage sites with angiopoietins (ANPs). Concurrent with ANP function, different ANGPTL proteins have shown variable regulatory activities in angiogenesis. Regardless of this overlap, the ANGPTL family does not bind to the tie 1 and tie 2 ANP receptors, indicating a different mechanism of action for the ANGPTL family. In the early stages of their discovery, the ANGPTL proteins were thought to be orphan ligands with different functional niches [5]. Nevertheless, recent studies have identified specific ligand-receptor interactions for ANGPTL family members. ANGPTL 2,5, and 7 have been shown to bind the immune-inhibitory human leukocyte immunoglobulin-like receptor B2 (LILRB2) to mediate human cord hematopoietic stem cell expansion [6]. ANGPTL3 and 4 have shown activity in lipid metabolism and regulation through lipoprotein lipase inhibition in different nutritional states of the body. Furthermore, studies have shown that ANGPTL1 exerts anti-angiogenic properties through its modulation of endothelial cells [7], while ANGPTL2 and ANGPTL4 play an integral part in the pro-angiogenic response to hypoxia exerted by different cancers [8,9]. Studies have shown that ANGPTL4 plays a diverse role in cancer development, progression, and dissemination. This has led to a lack of conclusive evidence and has led some researchers to postulate a conflicting role for ANGPTL4 in tumor biology.

ANGPTL4 expression has been associated with a worse prognosis in cervical cancer, gallbladder cancer, breast cancer, and pancreatic cancer [10–13]. However, newer studies seem to contradict these results. A study conducted by Cai et al revealed that overexpression of ANGPTL4 led to increased survival in triple negative breast cancer patients [14]. Results by Lin et al showing decreased osteosarcoma progression in the setting of ANGPTL4 overexpression further supports this notion. Furthermore, ANGPTL4 has been shown to both promote and inhibit metastasis based on different protein cleavage products and cancer types [15–17]. ANGPTL4 expression has been linked to chemotherapy resistance in pancreatic cancer, glioblastoma, and ovarian cancer [12,18,19], and is also linked to anoikis resistance and cancer progression [11,12,20,21].

This study aims to summarize the role of ANGPTL4 in the literature by performing a pan-cancer meta-analysis of observational studies and establish an overall role for ANGPTL4 in cancer by conducting a comprehensive bioinformatics investigation. This includes an analysis of prognostic outcomes, clinicopathological data, mutational burden, and genomic information to elucidate ANGPTL4’s central role across various cancer types. Furthermore, we identify cancers where ANGPTL4’s role in the TME, patient prognosis, and drug resistance is not yet explored, identifying potential new targets for treatment and biomarker selection.

## 2. Methods

The meta-analysis was conducted following the preferred reporting items for systematic review and meta-analysis (PRISMA) 2020 guidelines. The protocol can be found on PROSPERO with the ID number: CRD42024542298.

### 2.1. Research strategy

A comprehensive electronic literature search was performed on March 4th, 2024 using PubMed, Scopus, and Embase using the following terms (“ANGPTL” or “ANGPTL4” or “angiopoietin like protein 4”) AND (“role in” or “effect on”) AND (“metastasis” or “invasion” or “cancer” or “tumor” or “malignancy”) AND (“prognosis” or “survival”).

### 2.2. Inclusion and exclusion criteria

Exclusion criteria were as follows:

Studies that were published in the form of conference abstracts, letters, case reports, expert opinions, reviews, sequence data, studies focused on tumor cell lines or animal experiments, in addition to non-confirmed cancer patients, papers written in other languages, and papers with no access to original data. Furthermore, studies that did not split their cohort into high and low ANGPTL4 expression were also excluded.

Inclusion criteria were as follows

Studies that elucidated the association of ANGPTL4 with at least one clinicopathological or prognostic factors.

### 2.3. Data extraction and quality assessment

Studies were selected by two independent reviewers based on the inclusion and exclusion criteria. Disagreements were resolved by group discussion and consensus. The following information was collected from each study: author last name, publication year, country, sample type, detection method, criteria for patient categorization based on ANGPTL4 expression level, number of events and total sample size for clinicopathological characteristics in both high and low ANGPTL4 expression groups, the hazard ratio (HR) with its 95% confidence interval (CI) for overall survival (OS) and disease-free survival (DFS). For studies that did not report their HR we used the methodology proposed by Tierney et al to calculate the HR from Kaplan-Meier (KM) Curves. Engauge Digitizer 4.1 was used to extract time and event variables, and the calculation was based on the excel sheet provided in the article written by Tierney et al [22]. The Newcastle–Ottawa quality assessment scale (NOS) that considers selection, outcome, and comparability, was used to assess the quality of the studies for inclusion.

### 2.4. Statistical analysis

Meta-analysis was conducted using the R package “Meta” on R version 4.3.2 [23]. Pooled odds ratio (ORs) with 95% confidence intervals were used to assess correlation between ANGPTL4 expression and clinicopathological factors. Pooled HRs with 95% confidence intervals were used to quantify the correlation between ANGPTL4 expression and prognosis. In the case of non-significant heterogeneity (I^2 < 50%, P >  0.10) a fixed-effects model utilizing the Mantel-Haenszel method was used. If heterogeneity did arise (I^2 ≥ 50%, P ≤  0.10), a random effects model using the DerSimonian and Laird method was employed. Sensitivity analysis utilizing leave one out analysis was conducted using the same stipulations mentioned above. If a study shows significant alteration of the pooled effect size or p value, then the results upon its omission will be mentioned in the results section. Subgroups based on cancer type were implemented for OS. Publication bias was assessed using egger’s test and visual interpretation of the funnel plot. The trim and fill method (TAF) was employed if egger’s test showed significant publication bias (P < 0.05). The statistical significance cut off for both OR and HR results was set at P <  0.05.

### 2.5. TCGA validation and survival analysis

KM plots based on the upper and lower quartile of expression in The Cancer Genome Atlas (TCGA) cohort were extracted from the Gene Expression Profiling Interactive Analysis web tool (GEPIA2) (http://gepia2.cancer-pku.cn/#index) [24]. Multivariate Cox regression, on the same cohort, accounting for stage was conducted on TIMER2.0 (http://timer.cistrome.org) [25]. TIMER 2.0 is an interactive web-tool that facilitates the systematical analysis of immune infiltration using multiple different deconvolution tools, while also allowing analysis between gene expression and survival outcomes. We conducted the TCGA validation on all the following cancer types: Adrenocortical carcinoma (ACC), bladder urothelial carcinoma (BLCA), breast invasive carcinoma (BRCA), cervical squamous cell carcinoma and endocervical adenocarcinoma (CESC), cholangiocarcinoma (CHOL), colon adenocarcinoma (COAD), diffuse large B-cell lymphoma (DLBC), esophageal carcinoma (ESCA), glioblastoma multiforme (GBM), head and neck squamous cell carcinoma (HNSC), kidney chromophobe (KICH), kidney renal clear cell carcinoma (KIRC), kidney renal papillary cell carcinoma (KIRP), acute myeloid leukemia (LAML), brain lower grade glioma (LGG), liver hepatocellular carcinoma (LIHC), lung adenocarcinoma (LUAD), lung squamous cell carcinoma (LUSC), mesothelioma (MESO), ovarian serous cystadenocarcinoma (OV), pancreatic adenocarcinoma (PAAD), pheochromocytoma and paraganglioma (PCPG), prostate adenocarcinoma (PRAD), rectum adenocarcinoma (READ), sarcoma (SARC), skin cutaneous melanoma (SKCM), stomach adenocarcinoma (STAD), testicular germ cell tumors (TGCT), thyroid carcinoma (THCA), thymoma (THYM), uterine corpus endometrial carcinoma (UCEC), uterine carcinosarcoma (UCS), and uveal melanoma (UVM).

### 2.6. Drug sensitivity analysis

Drug sensitivity in relation to ANGPTL4 expression from the CTRP and GDSC databases was extracted from GSCAlite (https://guolab.wchscu.cn/GSCA/#/) [26]. GSCAlite is a comprehensive genomic, pharmacogenomic and immunogenomic analysis web-tool that utilizes the TCGA database for a plethora of analytical applications.

### 2.7. Mutation analysis

Alteration frequency of ANGPTL4 for all cancers along with alteration frequency of other genes in altered and non-altered ANGPTL4 groups was retrieved from cBioportal [27–29]. Further copy number variation (CNV) and single nucleotide variation (SNV) was conducted on GSCAlite [26].

### 2.8. Relationship between ANGPTL4 expression and immune infiltration

Immune infiltration was assessed via the ImmuCellAI [30] algorithm which measures the abundance of 24 immune cell types based on gene expression. The analysis was conducted using GSCAlite on 33 tumor types [26]. Correlation between ANGPTL4 and cancer-associated fibroblast (CAFs) infiltration was retrieved from TIMER2.0 [25].

### 2.9. Single-Cell Functional analysis of ANGPTL4

The Cancer single cell Atlas (CancerSEA) (http://biocc.hrbmu.edu.cn/CancerSEA/) is a comprehensive single cell RNA sequencing tool that assesses 14 cancer-related functional states in 41,900 cells across 25 cancer types [31]. We retrieved the functional states for 11 cancers in relation to ANGPTL4 expression.

### 2.10. Co-expressed genes and functional enrichment analysis

Co-expressed genes with their spearman’s correlation coefficients were retrieved from the cBioportal [27–29]. Gene set enrichment analysis (GSEA) for ANGPTL4 plus its co-expressed genes was conducted on enrichr [32–34]. The top 500 positively co-expressed and the top 500 negatively co-expressed genes were inserted into enrichr, and statistically significant (FDR <  0.05) cancer-related enrichment terms were retrieved. Plotting of the enriched terms was conducted on Python 3.11 using the package matplotlib. Important term quantifying metrics such as combined score and q values were used to show the importance of each term. Furthermore, gene set variation analysis (GSVA) pathway enrichment utilizing reverse phase protein assays from the TCPA database for each TCGA sample was conducted on GSCAlite. The top 100 positively and negatively co-expressed genes were used to elucidate ANGPTL4’s involvement in each of the pathways. Furthermore, the correlation between ANGPTL4 expression and TGFB response was extracted from TCGEx (https://tcgex.iyte.edu.tr/), which is a web analytical tool that allows for custom analysis between different variables in the TCGA datasets [35]. A Protein-to-protein interaction network was created using the STRING online database which links proteins based on their functional interactions [36].

## 3. Results

### 3.1. Search results

**Fig 1:**
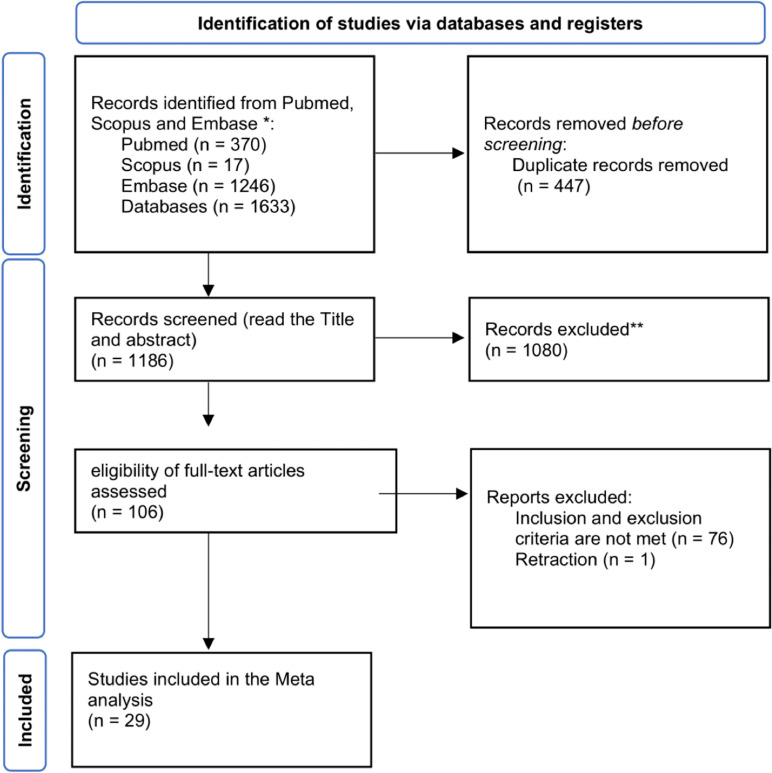
PRISMA flowchart.

The initial search strategy yielded 1633 papers from Pubmed, Embase, Scopus, and Web of Science. Duplicate detection identified 447 papers for omission, and a further 1080 papers were removed after title and abstract screening, leaving only 106 papers for full-text screening. Of those 106 papers, 30 papers met the inclusion criteria and were thus used in the Meta-Analysis (Fig 1).

### 3.2. Study characteristics

3268 cancer patients were included in our meta-analysis. All patients were divided based on high and low ANGPTL4 expression. Most studies were from China (n = 14) and Japan (n = 9). Other countries included were Egypt (n = 1), Norway (n = 1), Thailand (n = 1), Malaysia (n = 1), USA(n = 1), France (n = 1), and Korea(n = 1). Immunohistochemistry was the primary method of ANGPTL4 detection followed by ELISA and PCR. 16 different cancer types from 30 papers were included; Breast cancer (n = 5), colorectal cancer (n = 5), tongue squamous cell carcinoma (n = 3), gastric cancer (n = 2), esophageal squamous cell carcinoma (n = 2), hepatocellular carcinoma (n = 2), pancreatic cancer (n = 2), oral squamous cell carcinoma (n = 1), endometrial cancer (n = 1), cervical cancer (n = 1), renal cell carcinoma (n = 1), urothelial carcinoma (n = 1), gallbladder cancer (n = 1), prostate cancer (n = 1), non-small cell lung cancer (n = 1), and cholangiocarcinoma (n = 1). Study characteristics are summarized in S1 Table. In terms of quality assessment, all studies had an overall NOS score ranging from 6 to 9. All the details of QA are written in S2 Table.

### 3.3. Meta-analysis results

#### 3.3.1. Correlation between ANGPTL4 and clinicopathological characteristics.

**Fig 2:**
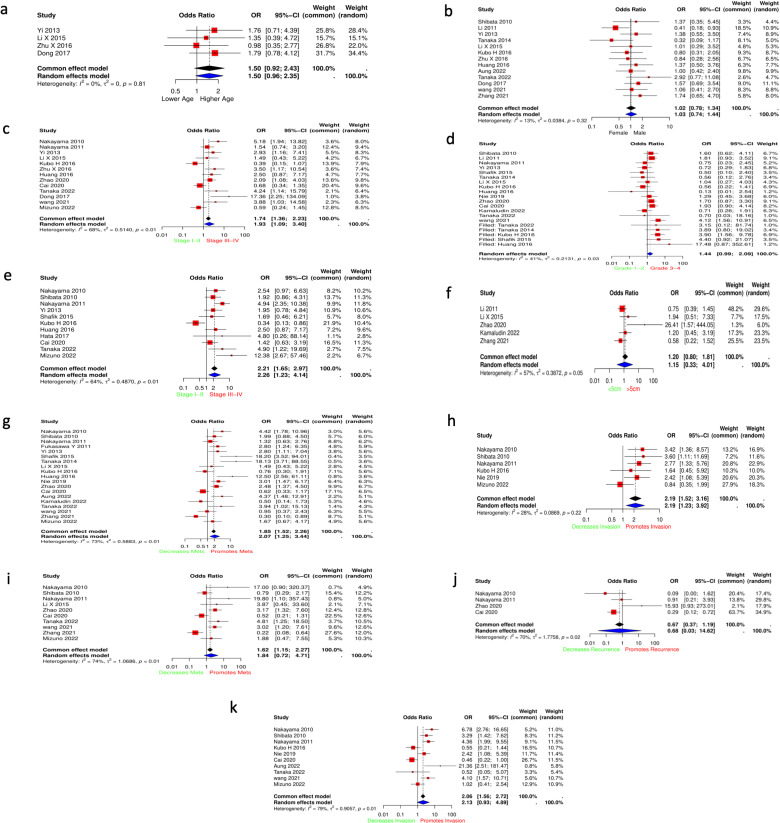
Forest plots for the clinicopathological factors. (A) Age (B) Gender (C) TNM staging (D) Histological differentiation (E) T staging (F) Tumor size (G) Lymph node metastasis (H) Lymphatic Invasion (I) Distant metastasis (J) Local recurrence (K) Vascular invasion.

**3.3.1.1 Age:** We included a total of 4 studies for age. Fig 2A shows that no significant correlation between age and ANGPTL4 expression was found (OR = 1.50, 95% CI: 0.92–2.43, P =  0.10). No significant heterogeneity was observed (I^2 =  0%, P =  0.81) and as such a fixed-effects model was employed.

**3.3.1.2 Gender:** A total of 13 studies were included for gender. No significant correlation between gender and ANGPTL4 expression was discovered (OR = 1.02, 95% CI: 0.78,1.34, P =  0.88). No significant heterogeneity was observed (I^2 =  13%, P =  0.32) (Fig 2B).

**3.3.1.3 TNM staging:** 13 papers reported results for TNM staging. A significant correlation was observed between high ANGPTL4 expression and more advanced TNM staging (stage III-IV) (OR =  1.93, 95% CI: 1.09–3.40, P =  0.027). The random effects model was employed due to significant heterogeneity between studies (I^2 =  68%, Tau^2 =  0.52, P =  0.0002) (Fig 2C).

**3.3.1.4. Histological differentiation:** A total of 14 studies reported histological differentiation/ tumor grade and were used in the analysis. A non-significant correlation between high ANGPTL4 expression and worse histological differentiation was observed (OR = 1.27, 95% CI: 0.984–1.63, P =  0.067). Low non-significant heterogeneity was discovered (I^2 =  41%, P =  0.03), and as such a fixed-effects model was utilized. However, significant publication bias was detected using egger’s test (intercept =  -2.22, P =  0.0083) (Table S3), and subsequent employment of the trim and fill method yielded a still non-significant association between ANGPTL4 expression and histological differentiation (OR = 1.44, 95% CI: 0.99–2.09, P =  0.056) (Fig 2D).

**3.3.1.5. T staging:** In terms of T staging, our analysis included 11 studies. Higher ANGPTL4 was significantly correlated with advanced stage (T3-T4) (OR =  2.26, 95% CI: 1.23–4.14, P =  0.01). Heterogeneity was significant (I^2 =  64%, Tau^2 =  0.487, P =  0.0018, which resulted in a random-effects model being employed (Fig 2E). It is to be noted that sensitivity analysis identified Kubo H 2016 as a major cause of heterogeneity. Removing KUBO H significantly fixed heterogeneity and increased the precision of the results (I^2 =  21%, Tau^2 =  0.0723). The new analysis strengthened the correlation between ANGPTL4 expression and advanced T staging (OR =  2.68, 95% CI: 1.23–4.14, P < 0.01) (Fig S2).

**3.3.1.6. Tumor size:** 5 studies were included for Tumor size. No significant correlation between ANGPTL4 and tumor size was discovered (OR =  1.15, 95% CI: 0.33–4.01, P =  0.77). A random effects model was used due to the presence of significant heterogeneity (I^2 =  57%, Tau^2 =  0.38, P =  0.054) (Fig 2F).

**3.3.1.7. Lymph node metastasis (LNM):** 19 studies were included in the analysis. Elevated ANGPTL4 was significantly associated with increased LNM (OR = 2.07, 95% CI:1.25–3.44, p < 0.0001). Substantial heterogeneity was discovered (I^2 = 73%, tau^2 = 0.5883, p < 0.0001), so a random effects model was employed (Fig 2G).

**3.3.1.8. Lymphatic invasion:** 6 studies were included in the analysis. Elevated ANGPTL4 was significantly associated with increased lymphatic invasion (OR =  2.19, 95% CI: 1.52–3.16, P <  0.0001). Observed heterogeneity was small and insignificant (tau^2 = 0.0869,I^2 =  28%, P =  0.22), so we employed a fixed effects model (Fig 2H).

**3.3.1.9. Distant metastasis:** 10 studies were included in the analysis for distant metastasis. Elevated ANGPTL4 was correlated with increased distant metastasis but did not achieve statistical significance (OR = 1.84, 95%CI: 0.72–4.71, p = 0.1747). Significant heterogeneity (I^2 = 74%, tau^2 = 1.0686, p < 0.0001) existed, and as such a random effects model was used (Fig 2I).

**3.3.1.10. Local recurrence:** A total of 4 studies reported local recurrence and were included in our analysis. Local recurrence was insignificantly associated with lower ANGPTL4 expression (OR =  0.68, 95% CI:0.03–14.62, P =  0.7159). Significant heterogeneity was present (Tau^2 =  1.7758, I^2 =  70%, P =  0.02), thus a random effects model was employed (Fig 2J).

**3.3.1.11. Vascular invasion:** A total of 10 studies were used in the analysis. Increased ANGPTL4 expression corresponded to increased vascular invasion, however statistical significance was not reached (OR =  2.13, 95% CI: 0.93–4.89, P =  0.07). Significant heterogeneity was discovered (Tau^2 =  0.9057, I^2 =  79%, P < 0.0001) and as such a random effects model was used (Fig 2K).

#### 3.3.2. Correlation between ANGPTL4 and survival.

**Fig 3:**
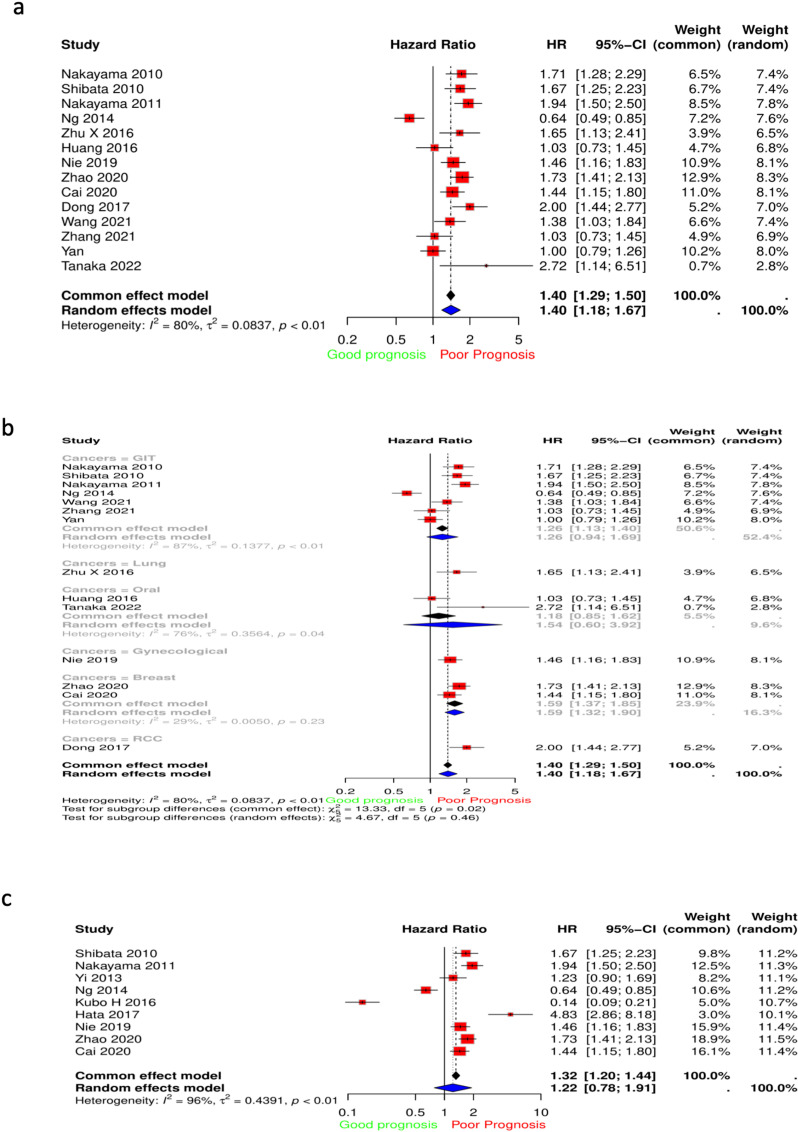
Forest plots for the meta-analysis of ANGPTL4 and survival. **Fig 3 shows the forest plots for prognostic outcomes. (A)** Forest plot for overall survival **(B)** Sub-group forest plot for overall survival **(C)** Forest plot for disease-free survival.

**3.3.2.1. ANGPTL4 expression and overall survival:** A total of 14 articles reported survival data and were included in the analysis. Up-regulation of ANGPTL4 was significantly associated with poor prognosis and worse overall survival (HR = 1.40, 95% CI: 1.29,1.50, P = 0.0001). Significant heterogeneity was discovered (Tau^2 =  0.0837, I^2 =  80%, P < 0.01), which necessitated a random-effects model (Fig 3A). Furthermore, a subgroup analysis based on cancer type was performed. Only 3 subgroups had 2 or more studies, thus allowing a pooled analysis. ANGPTL4 expression was linked to worse OS in GIT (HR =  1.26, 95% CI: 0.94–1.69) and Oral cancers (HR =  1.54, 95% CI: 0.60–3.92), however both subgroups did not achieve statistical significance. Heterogeneity was localized and significant to both GIT and Oral cancers, and as such the random effects model was used. On the other hand, subgrouping resolved heterogeneity in the breast cancer subgroup, and results show a significant association between ANGPTL4 expression and worse OS (HR =  1.59, 95% CI: 1.32–1.90) (Fig 3B).

**3.3.2.2. ANGPTL4 expression and Disease-free survival:** 9 total studies reported results for DFS and were included in our analysis. ANGPTL4 expression was linked to worse DFS, but did not reach statistical significance (HR =  1.22, 95% CI: 0.78–1.91, P =  0.3733). Heterogeneity was significant (I^2 =  96%, t^2 =  0.4391, P <  0.0001), and thus the random-effects model was utilized (Fig 3C). Further sensitivity analysis to assess heterogeneity revealed that Kubo H et al produced significant heterogeneity and influenced the significance of the pooled analysis. Omitting Kubo H 2016 from the analysis dropped heterogeneity to (I^2 =  89%, t^2 =  0.1464), and yielded a significant association between ANGPTL4 expression and worse DFS (HR =  1.56, 95% CI: 1.17–2.07, P <  0.01) (Fig S2).

#### 3.3.3. Publication bias and sensitivity analysis.

Publication bias was assessed using egger’s test and funnel plot assessment, and any significant findings were reported under each topic. All egger’s test values and funnel plots can be found in S3 Table and Fig S1, respectively. In terms of sensitivity analysis, any significant finding was reported under each topic. Fig S2 shows the leave-one-out plots utilized for the sensitivity analysis.

### 3.4. Bioinformatics results

#### 3.4.1. TCGA survival and expression validation.

**Fig 4:**
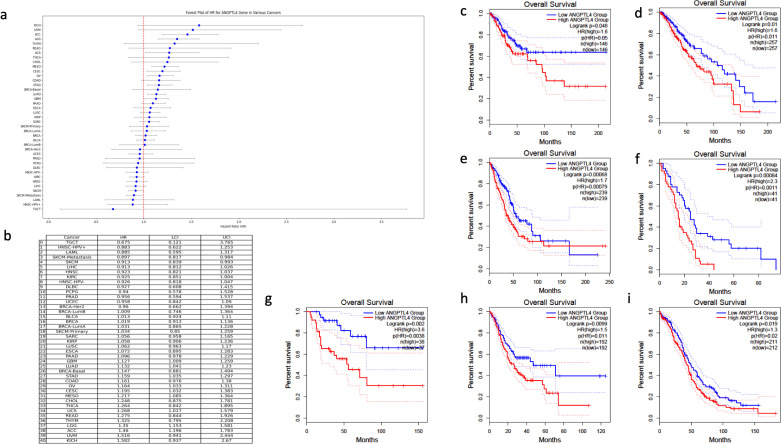
Multivariate cox regression and KM curves for survival based on ANGPTL4 expression. (A) Forest plot showing the multivariate HR for different cancers. (B) Table showing the multivariate HR values. (C) OS for CESC (D) OS for LGG (E) OS for LUAD (F) OS for Mesothelioma (G) OS for ACC (H) OS for STAD (I) OS for OV.

The multivariate cox regression accounting for stage revealed varying effects of ANGPTL4 expression on OS (Fig 4A). ANGPTL4 expression showed a protective effect on OS for SKCM (HR =  0.913, 95% CI: 0.839–0.993) and metastatic SKCM (HR =  0.897, 95%CI: 0.817–0.984). On the other hand, a harmful effect for ANGPTL4 expression was shown for GBM (HR =  1.127, 95% CI: 1.009–1.259), LUAD (HR =  1.132, 95%CI: 1.041–1.230), STAD (HR =  1.159, 95%CI: 1.035–1.297), OV (HR =  1.164, 95%CI: 1.033–1.311), CESC (HR =  1.195 95% CI: 1.032–1.383), MESO (HR =  1.217, 95%CI: 1.085–1.364), UCS (HR =  1.268, 95% CI: 1.017–1.579), LGG (HR =  1.35, 95% CI: 1.153–1.581), and ACC (HR =  1.46, 95% CI: 1.196–1.783) (Fig 4B). Kaplan-Meier curves were graphed for cancers in which ANGPTL4 had a significant effect on OS, this included CESC, LGG, LUAD, MESO, ACC, STAD, and OV (Fig 4C-4H).

#### 3.4.2. ANGPTL4 mutation analysis.

**Fig 5:**
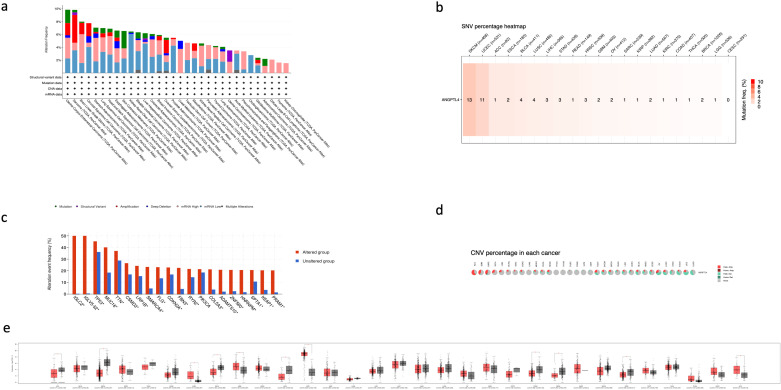
The landscape of ANGPTL4 alterations in cancer. **(A)** Shows the most frequent alterations of ANGPTL4 in different cancers including mutations, variants, amplifications, deep deletions, high or low expression, or the multiplicity of alterations. **(B)** Shows the percentage of deleterious ANGPTL4 mutations in each TCGA cancer. **(C)** Shows the alteration frequency of oncogenic genes between patients who have ANGPTL4 alteration and those who do not. **(D)** Shows the percentage of ANGPTL4 CNV’s per cancer. **(E)** Shows the expression of ANGPTL4 in normal and tumor tissues in different cancers. *  in Fig 5C represents a q value <  0.05.

Fig 5A shows a summary for genetic alterations in all TCGA cancers. UCEC, Sarcomas, and LGG had the 3 highest alteration frequencies with UCEC’s and LGG’s mostly experiencing increased mRNA expression, while sarcomas mostly had gene amplifications. We further explored deleterious single nucleotide variations of ANGPTL4. These include missense mutations, nonsense mutations, frame-shift mutations, and splice site mutations. Deleterious mutations were more common in SKCM and UCEC with 13 and 11 mutations respectively. All other cancer types experienced 0–4 deleterious mutations (Fig 5B). We also analyzed the proportion of copy number variations for each cancer type. ACC, GBM, Sarcomas, and KICH had the highest proportions of heterogeneous amplifications. In particular, sarcomas showed a relatively increased proportion of homogenous amplification in comparison to other cancers. LUAD, UCS, ESCA, STAD, LUSC, and OV showed the highest proportion of heterogenous deletion. Interestingly, OV cancers showed all 4 types of CNVs. Homogenous deletions were very rare across all cancer types (Fig 5D). Lastly, we elucidated the alteration frequency of other key oncogenic genes in relation to ANGPTL4 alteration frequency. High ANGPTL4 alteration groups experienced higher alteration percentages of key genes such as TP53, CDKN2A, PIK3CA, and COL5A3. IGLC2 and IGLV-53 both showed disproportionately high alteration frequency in the altered group, possibly elucidating a role for ANGPTL4 in B cell and plasma cell malignancies (Fig 5C). Pan-cancer gene expression analysis revealed that ANGPTL4 was differentially expressed and upregulated in DLBC, GBM, KIRC, and UCS, while being downregulated in ACC, BRCA, ESCA, KICH, PRAD, READ, and STAD (Fig 5E).

#### 3.4.3. Drug sensitivity analysis in relation to ANGPTL4 expression.

We used GSCAlite to assess the role of ANGPTL4 on drug sensitivity via both the GDSC and CTRP databases. The GDSC database showed that ANGPTL4 expression increased resistance to Navitoclax, vorinostat, and a couple other small molecule drugs, while increased expression sensitized drug response to important chemotherapeutics such as bleomycin and cisplatin (Fig S3A). On the other hand, the CTRP database’s top correlated drugs showed that increased ANGPTL4 expression conferred tumor resistance to commonly used chemotherapeutics such as chlorambucil, doxorubicin, and vincristine. Furthermore, ANGPTL4 expression increased resistance to six different histone deacetylase inhibitors including: apicidin, belinostat, entinostat, panobinostat, tacedinaline, and vorinostat (Fig S3B). These drugs are mainly used in hematological malignancies and could further implicate ANGPTL4’s involvement in hematological cancers.

#### 3.4.4. Immune infiltration and ANGPTL4.

**Fig 6:**
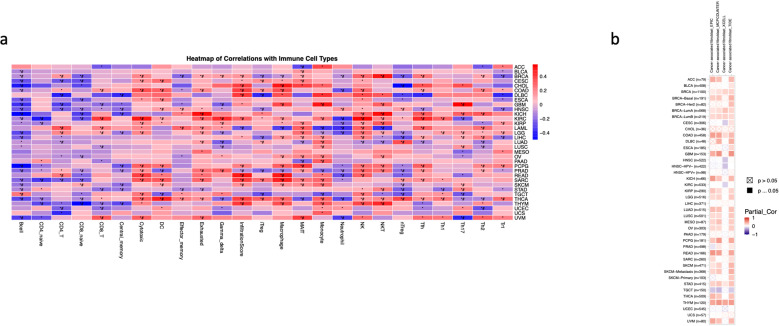
The correlation between immune infiltration and ANGPLT4 expression in cancer. **(A)** Shows the immune infiltration of 24 different immune cells based on ANGPTL4 expression, 17 of the 24 are different subsets of T cells. **(B)** Shows the infiltration of Cancer-associated fibroblasts based on ANGPTL4 expression. *  =  p <  0.05, # =  FDR <  0.05.

We carried out an immune infiltration assay of 24 different cell types across 33 different cancers utilizing the ImmuCellAI algorithm. Our analysis revealed that ANGPTL4 expression was significantly correlated with an increased immune infiltration score in: BRCA, CHOL, COAD, DLBC, KIRC, PCPG, SARC, SKCM, READ, THCA, AND THYM. On the other hand, lower immune infiltration scores were found in LAML and PRAD. A general trend of negative correlation between B cell (20/33 cancers), naïve CD8 T cell (11/33), Neutrophil (10/33), and central memory B cell (9/33) immune infiltration and ANGPTL4 expression was evident. However, ANGPTL4 expression was positively correlated with the immune infiltration of NK cells (14/33), Cytotoxic T cells (13/33), Macrophages (12/33), Exhausted T cells (11/33), and monocytes (11/33) (Fig 6A). We also carried out an immune infiltration assay of cancer associated fibroblasts (CAFs) using 4 different algorithms, a positive or negative correlation was only considered if similar results were uncovered in 3 or more algorithms. A positive correlation between ANGPTL4 expression and CAF infiltration was discovered in ACC, BRCA-Basal, BRCA-LumA, BRCA-LumB, COAD, DLBC, GBM, KICH, KIRP, LGG, LUAD, LUSC, MESO, OV, PCPG, READ, Metastatic SKCM, STAD, THCA, THYM, and UVM. Conversely, a negative association was only found in TGCT. (Fig 6B).

Type 1 regulatory T cells (Tr1), Type 2 helper T cells (Th2), Type 17 helper T cells (Th17), Type 1 helper T cells (Th1), Follicular T cells (Tfh), T regulatory cells (nTreg), Natural killer T cells (NKT), Natural killer cells (NK), Mucosal-associated invariant T cells (MAIT).

#### 3.4.5. Single cell functional enrichment.

**Fig 7:**
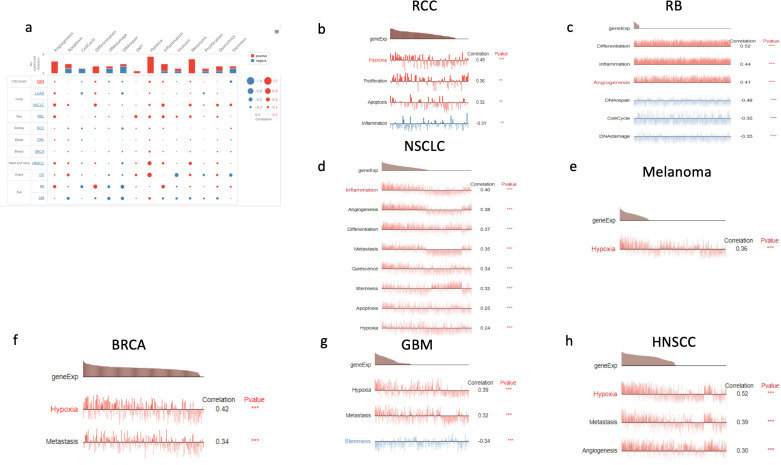
Correlation between ANGPTL4 and cancer cell functional state at the single cell level. **(A)** Dot plot graph showing the correlation between 11 cancer types and 14 different pathways based on ANGPTL4 expression. **(B)** Shows the single cell functional enrichment of RCC. **(C)** Shows the single cell functional enrichment for RB. **(D)** Shows the single cell functional enrichment of NSCLC. **(E)** Shows the single cell functional enrichment for melanoma. **(F)** Shows the single cell functional enrichment for BRCA. **(G)** Shows the single cell functional enrichment for GBM. **(H)** Shows the single cell functional enrichment for HNSCC. Legend: *** p<=0.001; ** p<=0.01; *  p<=0.05.

To elucidate a more specific role for ANGPTL4 in different cancer types, we carried out single cell functional enrichment analysis for 11 different cancer types. ANGPTL4 expression showed a mostly positive correlation with angiogenesis, hypoxia, and metastasis, while showing a mostly negative correlation with DNA repair, cell cycle, and stemness (Fig 7A). Functional enrichment of RCC showed that ANGPTL4 increased hypoxia, proliferation, and apoptosis, while decreasing inflammation. In RB, ANGPTL4 was associated with increased differentiation, inflammation, and angiogenesis, while also being linked to decreased DNA repair, cell cycle progression, and DNA damage. In NSCLC, increased ANGPTL4 expression was positively correlated with inflammation, angiogenesis, differentiation, metastasis, quiescence, stemness, apoptosis, and hypoxia. ANGPTL4 expression was associated with increased hypoxia in Melanoma, and increased hypoxia and metastasis in BRCA. In GBM, ANGPTL4 was correlated with increased hypoxia and metastasis, and decreased stemness. Finally, ANGPTL4 expression in HNSCC was correlated with increased hypoxia, metastasis, and angiogenesis (Fig 7B-7H).

#### 3.4.6. Protein-protein interaction network, gene set enrichment analysis, and pathway enrichment.

**Fig 8:**
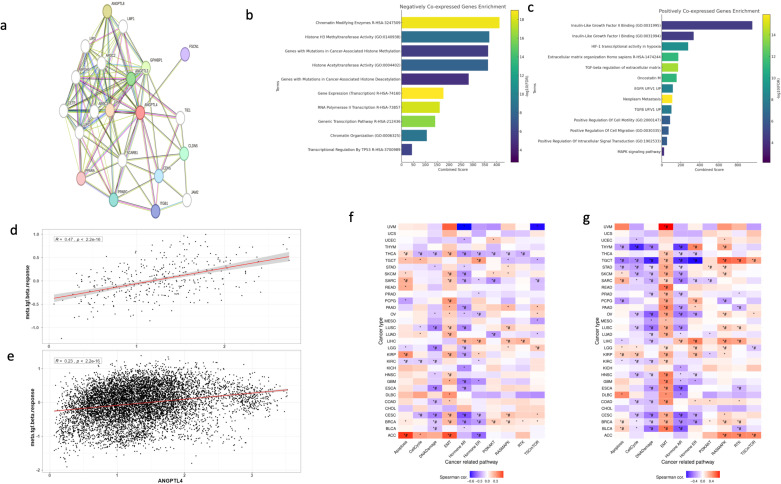
Protein-Protein Interaction Network, gene set enrichment analysis, and co-expressed genes with ANGPTL4 in cancer. **(A)** Shows a PPI network for ANGPTL4 highlighting ANGPTL4’s interaction with other proteins. **(B)** Shows the GSEA of negatively co-expressed genes. **(C)** Shows the GSEA of positively co-expressed genes. **(D)** Shows the correlation between ANGPTL4 and TGFB response in metastatic cells. **(E)** Shows the correlation between ANGPTL4 and TGFB response in primary tumor cells. **(F)** Shows the GSVA pathway enrichment for the genes negatively co-expressed with ANGPTL4. **(G)** Shows the GSVA pathway enrichment for the genes positively co-expressed with ANGPTL4. *  =  p <  0.05, # =  FDR <  0.05.

We aimed to investigate the interactions of ANGPTL4 with other proteins by constructing a protein-protein interaction (PPI) network via STRING. The ANGPTL4 PPI network revealed numerous interactions with two subtypes of proteins: Proteins involved in lipid metabolism and proteins involved in cell-cell junctions. In terms of metabolism related proteins, ANGPTL4 was linked to ANGPTL3, LPL, APOC2, APOC3, SCARB1, PPARA, and PPARG. On the other hand, ANGPTL4 showed interactions with proteins involved in junctional and ECM integrity. This included CDH5, CLDN5, ITGB1, and JAM2 (Fig 8A). Furthermore, to elucidate the role of ANGPTL4 in cancer we conducted GSEA on the top 500 positively and negatively co-expressed genes. GSEA on negatively co-expressed genes showed terms mostly related to DNA structure and transcription such as chromatin modifying enzymes, transcriptional regulation by TP53, histone H3 methyltransferase activity, histone acetyltransferase activity, chromatin organization, genes with mutations in cancer-associated histone deacetylation and methylation (Fig 8B). Conversely, the positively co-expressed genes revealed terms like TGF-beta regulation of extracellular matrix, positive regulation of cell motility, positive regulation of intracellular signal transduction, positive regulation of cell migration, extracellular matrix organization, neoplasm metastasis, HIF-1 transcriptional activity in hypoxia, and MAPK signaling pathway (Fig 8C). In order to elucidate the relationship between ANGPTL4 and TGFB, a correlation analysis was conducted. ANGPTL4 expression was positively correlated with an increased TGFB response in primary tumor cells (r =  0.23, p <  0.001) and metastatic tumor cells (r =  0.47, p <  0.001) (Fig 8D and 8E). Lastly, using the top 100 positively and negatively co-expressed genes, we conducted a GSVA analysis to explore the impact of ANGPTL4 on cancer related pathways. The negatively co-expressed genes showed varying involvement in cancer, with no strong trend observed. Epithelial to mesenchymal transition was the pathway most positively correlated with ANGPTL4 (n = 11), and the AR pathway was the most negatively correlated pathway (n = 11) (Fig 8F). Positively co-expressed genes showed a strong positive correlation with epithelial to mesenchymal transition in 23 different cancers, while also exhibiting a negative correlation with DNA damage (n = 17), cell cycle pathways (n = 10), and the AR pathway (n = 14) (Fig 8G).

## 4. Discussion

ANGPTL4 is a secreted glycoprotein with 3 different cleavage products that have different functions. Full-length ANGPTL4 (fANGPTL4) is a 45–65 kDa glycoprotein secreted by adipose tissue [37]. fANGPTL4’s cleavage is completely tissue dependent. The C-fragment of ANGPTL4 (cANGPTL4) has been shown to possess multiple pro-tumorigenic activities such as promoting metastasis, tumor growth, and facilitating angiogenesis. On the other hand, the n-fragment of ANGPTL4 (nANGPTL4) has been shown to inhibit metastasis and tumor growth [37–39]. This tissue-dependent cleavage is the main facilitator of ANGPTL4’s dual role. Recent studies have shown that tumors mainly secrete fANGPTL4 and cANGPTL4 in the tumor micro-environment (TME). This increased expression in the TME correlates to cancer progression in colon carcinoma, squamous cell carcinoma, and melanoma patient samples. Furthermore, cANGPTL4 is highly expressed in epithelial tumors such as SCC, breast tumors, basal cell carcinomas, melanomas, and is linked to cancer progression. Tracing ANGPTL4 fragments also revealed that nANPGTL4 was the most abundant circulating form and was inversely correlated to disease progression [38].

In the current study we provide evidence that ANGPTL4 plays a harmful role in multiple cancers. Our meta-analysis revealed that there was a significant association between ANGPTL4 and worse TNM stage, T stage, lymphatic invasion, and lymph node metastasis. Moreover, ANGPTL4 resulted in worse OS when combining all cancer types. This effect was consistent when subgrouping for cancer type, as ANGPTL4 was linked to worse overall survival in breast cancer, gastrointestinal cancers, and oral cancers, despite not reaching statistical significance in the latter two. We further solidify ANGPTL4’s involvement in lymphatic cancer dissemination, a topic that remains contentious. Several studies suggest that ANGPTL4 inhibits lymphatic metastasis [38–40], yet our pooled analysis shows that increased ANGPTL4 expression is associated with increased lymphatic invasion and metastasis. However, our pooled analysis also shows that ANGPTL4 expression is not significantly linked to systemic metastasis. This is in congruence with the literature as multiple studies have shown that ANGPTL4 does not appear to play an independent role in metastasis [40–42]. Instead, the role of ANGPTL4 in metastasis appears to be TGF-B dependent [43–45]. Padua et al showed that TGF-B prepared breast cancer cells for metastasis by inducing ANGPTL4 expression. We further expand on this point by showing that ANGPTL4 is more positively correlated to TGF-B response in metastatic tumors in general when compared to primary tumors, highlighting the TGF-b/ANGPTL4 relationship in metastatic tumors [43]. Furthermore, GSEA of positively co-expressed genes yielded terms like TGF-beta regulation of extracellular matrix, positive regulation of cell motility, positive regulation of cell migration, and neoplasm metastasis. Other pathways through which ANGPTL4 has exhibited a tendency to initiate metastasis includes the FGF19/ANGPTL4 axis. Fan et al showed that FGF19 promoted hepatic stellate cells to transform into ANGPTL4 releasing CAFs. This promoted colorectal cancer cell motility and liver metastasis in-vitro and in-vivo. However, treatment of the same in-vitro cell lines with ANGPTL4 did not increase FGF19 expression. Showing that ANGPTL4 plays a role in systemic metastasis as a part of larger signaling pathways, and not independently. This mediator action of ANGPTL4 presents a suitable drug target that should be explored more thoroughly, as both studies mentioned above show that ANGPTL4 knock down results in decreased tumor migration and motility [46].

CAFs are an integral part of a tumor’s microenvironment and are usually the most abundant stromal cell type. They play a crucial role in promoting tumor growth, metastasis and treatment resistance. ANGPTL4 has been shown to promote the pro-tumorigenic effect of CAFs in a murine model of breast cancer by inducing STAT3, a transcription factor known for its role in EMT, angiogenesis, and inhibiting apoptosis [47]. Moreover, a study by Xiong et al showed that CAFs promote chemoresistance to taxane based therapy in prostate cancer cells by secreting ANGPTL4, which in turn binds to IQGAP1 activating the Raf-MEK-ERK-PGC1a axis. This increases oxidative phosphorylation and mitochondrial biogenesis, aiding in cancer cell proliferation and survival [48]. ANGPTL4 was also differentially expressed in gallbladder cancer associated fibroblasts when compared to normal fibroblasts, as well as being co-localized with protein-1 and a-smooth muscle actin, reflecting the strong co-expression between ANGPTL4 and fibroblast markers [10]. We show that increased ANGPTL4 expression is associated with CAF infiltration in 21 different cancer types, elucidating that ANGPTL4 expression is a strong predictor of CAF infiltration, and providing another explanation to why TME expression of ANGPTL4 correlates to poor prognosis. Furthermore, ANGPTL4 plays a role in ovarian cancer induced mesothelial cell migration and mesothelial to mesenchymal transformation, alongside promoting mesothelial cell induced ovarian cancer proliferation, migration, cell adhesion, and invasion [49]. Our findings necessitate the exploration of the role of ANGPTL4 in different cancers such as glioblastoma, lower grade glioma, renal cell carcinoma, lung adenocarcinoma, lung squamous cell carcinoma, ovarian carcinoma, and rectal adenocarcinoma, as targeting ANGTPL4 could be of therapeutic benefit. This was shown again by Xiong et al, where targeting IQGAP1, a receptor for ANGPTL4, reversed ANGPTL4 dependent prostate cancer chemoresistance to docetaxel [48]. This corroborates the fact that ANGPTL4 is an important signaling protein between tumor cells and its stromal environment, facilitating many of the detrimental effects of cancer.

Furthermore, our single-cell functional analysis of ANGPTL4 revealed its important involvement in response to hypoxia and angiogenesis. Hypoxia is a key modulator of the TME that creates a harsh environment where more malignant cancer cells thrive. In response to hypoxia, key transcription factors such as HIF-1a and HIF-2a are upregulated and go on to increase the expression of genes involved in the metastatic cascade [50,51]. HIF-1 appears to regulate the expression of ANGPTL4 as one of its key products. C-ANGPTL4 as a protein product and ANGPTL4 at the genomic level look to be involved in the pro-angiogenic response to hypoxia mediated by HIF-1. Along with that, HIF-1 induced ANGPTL4 appears to promote anoikis resistance and tumor growth in scirrhous gastric cancer [52]. We show that ANGPTL4 is very strongly correlated with hypoxia in RCC, Melanoma, NSCLC, BRCA, GBM, and head and neck squamous cell carcinoma.

This is particularly interesting in RCC, who’s pathogenesis has long been known to depend on the biallelic loss of the Von-hippel lindau (VHL) gene. VHL functions as the recognition subunit of HIF-1a and HIF-2a in an E3-Ubiquitin ligase degradation system. Loss of VHL leads to the overexpression of HIF-1a and HIF-2a with the overexpression of their downstream targets [53]. Our analysis showed that increased expression of ANGPTL4 is positively correlated with hypoxia and proliferation in RCC, alongside conferring a poorer OS. Moreover, ANGPTL4 expression showed a negative correlation with inflammation. Yet, our immune analysis showed an increase in TH1, Tfh, NKT, NK, Monocyte, Macrophage, Cytotoxic and CD8 + T cells, and CD4 + T cells. Of interest, ANGPTL4 expression also correlated to increased infiltration of Exhausted cytotoxic T cells and decreased infiltration of neutrophils, naïve CD8 + T cells, and B cells, possibly explaining why ANGPTL4 is negatively correlated to inflammation yet has an overall positive correlation with immune infiltration in RCC. A recent study by Jin et al showed elevated expression of ANGPTL4 is only harmful in RCC tumors who possess VHL mutations. In fact, ANGPTL4 exhibited a tumor suppressive effect in VHL WT tumors by inhibiting lysosomal acid lipase [54]. Overall, this shows how ANGPTL4’s role is context dependent especially among different cellular states such as hypoxia. Clinical trials aiming to validate whether ANGPTL4 is a suitable hypoxic biomarker for RCC is currently recruiting patients and is set to finish by the end of 2025 (NCT05214885).

Our GSEA results further support this continuum, revealing terms like HIF-1α transcriptional activity in hypoxia associated with positively co-expressed genes. This further supports the notion that hypoxic TME’s are associated with increased ANGPTL4 expression.

We further performed a multivariate Cox regression analysis to solidify ANGPTL4’s role in cancer prognosis. Most significant findings indicate that ANGPTL4 is associated with poorer overall survival (OS) in cancer patient except melanoma and metastatic melanoma which showed that ANGPTL4 is protective, however, this conflicts with previously reported literature that shows ANGPTL4 predicts worse prognosis in melanoma [55,56]. It has been reported that ANGPTL4 plays a role in epithelial-to-mesenchymal transition (EMT) in melanoma and ovarian cancer by regulating the ERK1/2 signaling pathways [57,58]. Liao et al. further cemented the role of ANGPTL4 in driving EMT by discovering a novel extracellular matrix reorganizing signature that was mainly coordinated by ANGPTL4. Furthermore, ANGPTL4 inhibition via antisense oligonucleotides or c-ANGPTL4 antibodies lead to dose-dependent attenuation of EMT markers in a 3D-tumor model [59]. This is further supported by our results where GSVA pathway enrichment showed that genes positively co-expressed with ANGPTL4 were positively correlated to EMT in 23 different cancers. These included cancers such as uveal melanoma, stomach adenocarcinomas, lung squamous cell carcinoma, and lung adenocarcinoma.

Our analysis also highlighted that increased ANGPTL4 expression was correlated to increased resistance to histone deacetylase inhibitors (HDACs). GSEA of the negatively co-expressed genes also showed enriched terms such as histone acetyltransferase activity and genes with mutations in cancer-associated histone deacetylation. This implies that ANGPTL4 is negatively co-expressed with genes involved in histone deacetylation, possibly acting as a marker of treatment resistance. On the other hand of the resistance scale, our results indicated that ANGPTL4 expression was correlated with sensitivity to antitumor antibiotics and alkylating agents such as bleomycin and cisplatin, respectively. Evidence in the literature with regards to whether ANGPTL4 is associated with chemoresistance to platinum alkylating agents varies. Two studies showed that increased ANGPTL4 was associated with increased resistance to both cisplatin and carboplatin in ovarian cancer. Zhou et al revealed that ANGPTL4 released by adipocytes in the context of mice fed a high fat diet increased resistance to carboplatin, and interfering with ANGPTL4 secretion from adipocytes was able to increase carboplatin sensitivity [60]. A study by Kolb et al proved that increased activation of the inflammatory markers NLRC4 and IL-1b lead to a rise in ANGPTL4 secretion in primary adipocytes [61] showing a possible mechanism through which inflammation-mediated secretion of ANGPTL4 could lead to the progression of cancer. Li et al also showed that ANGPTL4 mediates resistance to cisplatin via the TAZ/ANGPTL4/SOX2 axis [62]. Yet, McEvoy et al showed that ANGPTL4 expression was increased in partial responders to cisplatin, in comparison to non-responders. This conflicting evidence might be due to the different mechanisms of ANGPTL4 expression. We also hypothesize that the increased expression of ANGPTL4 in patients who respond to cisplatin could be paradoxical, where responders were undergoing a form of adaptation by becoming more hypoxic, a mechanism known to induce cisplatin chemoresistance [63]. Further research utilizing ANGPTL4 knock down experiments in vitro and in vivo prior to treatment with cisplatin is needed to solidify ANGPTL4’s role in mediating alkylating agent resistance.

Overall, our study demonstrates that ANGPTL4 exerts a detrimental impact at the genomic level by influencing tumor migration, EMT, cell-cell communication within the TME, and immune infiltration. The involvement of ANGPTL4 for every cancer is highlighted in Table S4. Some important limitations must be addressed. First, most studies did not mention which fragment of ANGPTL4 they measured, and this could have been a major source of heterogeneity observed in the meta-analysis. However, given that most studies used the same sample type (tumor tissue samples), we can assume that most studies measured either fANGPTL4 or cANGPTL4 due to their relative abundance in the TME in comparison to nANGPTL4. Other possible causes of heterogeneity could be previous lines of therapy or purely due to the different role of ANGPTL4 in different cancer types. Both of which were latter elucidated in the bioinformatics analysis. Secondly, some survival data including HRs and 95% confidence intervals were extracted from KM curves, and thus may not be completely accurate. We tried to mitigate the potential bias that this might introduce by following the methodology produced by Tierney et al [22]. Lastly, since all our data, whether from online databases or observational studies, was retrospective, it may present an incomplete picture and introduce potential bias into our results.

## Supporting Information

S1 Fig Meta analysis funnel plots. Figure S1 shows the funnel plots for all the pooled outcomes. (A) Age (B) Gender (C) TNM staging (D) Histological differentiation (E) T staging (F) Tumor size (G) Lymph node metastasis (H) Lymphatic Invasion (I) Distant metastasis (J) Local recurrence (K) Vascular invasion (L) Overall Survival (M) Disease-Free Survival.
(PDF)

S2 Fig Sensitivity analysis for all outcomes. Figure S2 shows leave one out plots that represent the sensitivity analysis. (A) Age (B) Gender (C) TNM staging (D) Histological differentiation (E) T staging (F) Tumor size (G) Lymph node metastasis (H) Lymphatic Invasion (I) Distant metastasis (J) Local recurrence (K) Vascular invasion (L) Overall Survival (M) Disease-Free Survival.
(PDF)

S3 Fig Drug sensitivity analysis for all cancers. Figure S3 shows two dot plots that represent the correlation between ANGPTL4 expression and drug sensitivity. A positive correlation indicates that increased ANGPTL4 expression leads to increased drug resistance, and vice versa. (A) Shows the drug resistance profile from the GDSC. (B) Shows the drug resistance profile from the CTRP.
(PDF)

S1 Table Characteristics for all studies included in the analysis. Characteristics of all studies included in the analysis including survival outcomes and the presence of clinicopathological outcomes [64–87].
(DOCX)

S2 Table Quality assessment of all studies. Table S2 shows the New Ottawa Scale based quality assessment for all studies.
(DOCX)

S3 Table Egger’s tests. Table S3 shows the egger’s results for all the meta-analytic outcomes.
(DOCX)

S4 Table Summary of ANGPTL4’s role in every analyzed cancer. (XLSX)

S1 File Data Extraction File. (XLSX)

## References

[1] Deo SVS, Sharma J, Kumar S. GLOBOCAN 2020 report on global cancer burden: challenges and opportunities for surgical oncologists. Ann Surg Oncol. 2022;29(11):6497–500. doi: 10.1245/s10434-022-12151-6 35838905

[2] Novikov NM, Zolotaryova SY, Gautreau AM, Denisov EV. Mutational drivers of cancer cell migration and invasion. Br J Cancer. 2021;124(1):102–14. doi: 10.1038/s41416-020-01149-0 33204027 PMC7784720

[3] Hanahan D, Weinberg RA. Hallmarks of cancer: the next generation. Cell. 2011;144(5):646–74. doi: 10.1016/j.cell.2011.02.013 21376230

[4] Santulli G. Angiopoietin-like proteins: a comprehensive look. Front Endocrinol (Lausanne). 2014;5:4. doi: 10.3389/fendo.2014.00004 24478758 PMC3899539

[5] Kim I, Moon SO, Koh KN, Kim H, Uhm CS, Kwak HJ, et al. Molecular cloning, expression, and characterization of angiopoietin-related protein. Angiopoietin-related protein induces endothelial cell sprouting. J Biol Chem. 1999;274(37):26523–8. doi: 10.1074/jbc.274.37.26523 10473614

[6] Zheng J, Umikawa M, Cui C, Li J, Chen X, Zhang C, et al. Inhibitory receptors bind ANGPTLs and support blood stem cells and leukaemia development. Nature. 2012;485(7400):656–60. doi: 10.1038/nature11095 22660330 PMC3367397

[7] Dhanabal M, LaRochelle WJ, Jeffers M, Herrmann J, Rastelli L, McDonald WF, et al. Angioarrestin: an antiangiogenic protein with tumor-inhibiting properties. Cancer Res. 2002;62(13):3834–41. 12097297

[8] Wang X, Hu Z, Wang Z, Cui Y, Cui X. Angiopoietin-like protein 2 is an important facilitator of tumor proliferation, metastasis, angiogenesis and glycolysis in osteosarcoma. Am J Transl Res. 2019;11(10):6341–55. 31737187 PMC6834488

[9] Chédeville AL, Lourdusamy A, Monteiro AR, Hill R, Madureira PA. Investigating glioblastoma response to hypoxia. Biomedicines. 2020;8(9):310. doi: 10.3390/biomedicines8090310 32867190 PMC7555589

[10] Wang F-T, Li X-P, Pan M-S, Hassan M, Sun W, Fan Y-Z. Identification of the prognostic value of elevated ANGPTL4 expression in gallbladder cancer-associated fibroblasts. Cancer Med. 2021;10(17):6035–47. doi: 10.1002/cam4.4150 34331381 PMC8419759

[11] Zhao J, Liu J, Wu N, Zhang H, Zhang S, Li L, et al. ANGPTL4 overexpression is associated with progression and poor prognosis in breast cancer. Oncol Lett. 2020;20(3):2499–505. doi: 10.3892/ol.2020.11768 32782569 PMC7399784

[12] Gordon ER, Wright CA, James M, Cooper SJ. Transcriptomic and functional analysis of ANGPTL4 overexpression in pancreatic cancer nominates targets that reverse chemoresistance. BMC Cancer. 2023;23(1):524. doi: 10.1186/s12885-023-11010-1 37291514 PMC10251551

[13] Nie D, Zheng Q, Liu L, Mao X, Li Z. Up-regulated of angiopoietin-like protein 4 predicts poor prognosis in cervical cancer. J Cancer. 2019;10(8):1896–901. doi: 10.7150/jca.29916 31205547 PMC6547978

[14] Cai Y-C, Yang H, Wang K-F, Chen T-H, Jiang W-Q, Shi Y-X. ANGPTL4 overexpression inhibits tumor cell adhesion and migration and predicts favorable prognosis of triple-negative breast cancer. BMC Cancer. 2020;20(1):878. doi: 10.1186/s12885-020-07343-w 32928141 PMC7489026

[15] Bajwa P, Kordylewicz K, Bilecz A, Lastra RR, Wroblewski K, Rinkevich Y, et al. Cancer-associated mesothelial cell-derived ANGPTL4 and STC1 promote the early steps of ovarian cancer metastasis. JCI Insight. 2023;8(6):e163019. doi: 10.1172/jci.insight.163019 36795484 PMC10070116

[16] Hübers C, Abdul Pari AA, Grieshober D, Petkov M, Schmidt A, Messmer T, et al. Primary tumor-derived systemic nANGPTL4 inhibits metastasis. J Exp Med. 2023;220(1):e20202595. doi: 10.1084/jem.20202595 36269299 PMC9595206

[17] Izraely S, Ben-Menachem S, Sagi-Assif O, Meshel T, Marzese DM, Ohe S, et al. ANGPTL4 promotes the progression of cutaneous melanoma to brain metastasis. Oncotarget. 2017;8(44):75778–96. doi: 10.18632/oncotarget.19018 29100268 PMC5652662

[18] Tsai Y-T, Wu A-C, Yang W-B, Kao T-J, Chuang J-Y, Chang W-C, et al. ANGPTL4 induces TMZ resistance of glioblastoma by promoting cancer stemness enrichment via the EGFR/AKT/4E-BP1 cascade. Int J Mol Sci. 2019;20(22):5625. doi: 10.3390/ijms20225625 31717924 PMC6888274

[19] Li C, Wang Q, Luo Y, Xiang J. TAZ regulates the cisplatin resistance of epithelial ovarian cancer cells via the ANGPTL4/SOX2 axis. Anal Cell Pathol (Amst). 2022;2022:5632164. doi: 10.1155/2022/5632164 36247876 PMC9553699

[20] Zhu P, Tan MJ, Huang R-L, Tan CK, Chong HC, Pal M, et al. Angiopoietin-like 4 protein elevates the prosurvival intracellular O2(-):H2O2 ratio and confers anoikis resistance to tumors. Cancer Cell. 2011;19(3):401–15. doi: 10.1016/j.ccr.2011.01.018 21397862

[21] Liao Y-H, Chiang K-H, Shieh J-M, Huang C-R, Shen C-J, Huang W-C, et al. Epidermal growth factor-induced ANGPTL4 enhances anoikis resistance and tumour metastasis in head and neck squamous cell carcinoma. Oncogene. 2017;36(16):2228–42. doi: 10.1038/onc.2016.371 27797381 PMC5415642

[22] Tierney JF, Stewart LA, Ghersi D, Burdett S, Sydes MR. Practical methods for incorporating summary time-to-event data into meta-analysis. Trials. 2007;8:16. doi: 10.1186/1745-6215-8-16 17555582 PMC1920534

[23] Balduzzi S, Rücker G, Schwarzer G. How to perform a meta-analysis with R: a practical tutorial. Evid Based Ment Health. 2019;22(4):153–60. doi: 10.1136/ebmental-2019-300117 31563865 PMC10231495

[24] Tang Z, Kang B, Li C, Chen T, Zhang Z. GEPIA2: an enhanced web server for large-scale expression profiling and interactive analysis. Nucleic Acids Res. 2019;47(W1):W556–60. doi: 10.1093/nar/gkz430 31114875 PMC6602440

[25] Li T, Fu J, Zeng Z, Cohen D, Li J, Chen Q, et al. TIMER2.0 for analysis of tumor-infiltrating immune cells. Nucleic Acids Res. 2020;48(W1):W509–14. doi: 10.1093/nar/gkaa407 32442275 PMC7319575

[26] Liu CJ, Hu FF, Xie GY, Miao YR, Li XW, Zeng Y, et al. GSCA: an integrated platform for gene set cancer analysis at genomic, pharmacogenomic, and immunogenomic levels. Brief Bioinform. 2022;bbac558. 10.1093/bib/bbac558 36549921

[27] de Bruijn I, Kundra R, Mastrogiacomo B, Tran TN, Sikina L, Mazor T, et al. Analysis and visualization of longitudinal genomic and clinical data from the AACR project GENIE biopharma collaborative in cBioPortal. Cancer Res. 2023;83(23):3861–7. doi: 10.1158/0008-5472.CAN-23-0816 37668528 PMC10690089

[28] Gao J, Aksoy BA, Dogrusoz U, Dresdner G, Gross B, Sumer SO, et al. Integrative analysis of complex cancer genomics and clinical profiles using the cBioPortal. Sci Signal. 2013;6(269):pl1. doi: 10.1126/scisignal.2004088 23550210 PMC4160307

[29] Cerami E, Gao J, Dogrusoz U, Gross BE, Sumer SO, Aksoy BA, et al. The cBio cancer genomics portal: an open platform for exploring multidimensional cancer genomics data. Cancer Discov. 2012;2(5):401–4. doi: 10.1158/2159-8290.CD-12-0095 22588877 PMC3956037

[30] Miao Y-R, Zhang Q, Lei Q, Luo M, Xie G-Y, Wang H, et al. ImmuCellAI: a unique method for comprehensive T-cell subsets abundance prediction and its application in cancer immunotherapy. Adv Sci (Weinh). 2020;7(7):1902880. doi: 10.1002/advs.201902880 32274301 PMC7141005

[31] Yuan H, Yan M, Zhang G, Liu W, Deng C, Liao G, et al. CancerSEA: a cancer single-cell state atlas. Nucleic Acids Res. 2019;47(D1):D900–8. doi: 10.1093/nar/gky939 30329142 PMC6324047

[32] Chen EY, Tan CM, Kou Y, Duan Q, Wang Z, Meirelles GV, et al. Enrichr: interactive and collaborative HTML5 gene list enrichment analysis tool. BMC Bioinformatics. 2013;14:128. doi: 10.1186/1471-2105-14-128 23586463 PMC3637064

[33] Kuleshov MV, Jones MR, Rouillard AD, Fernandez NF, Duan Q, Wang Z, et al. Enrichr: a comprehensive gene set enrichment analysis web server 2016 update. Nucleic Acids Res. 2016;44(W1):W90-7. doi: 10.1093/nar/gkw377 27141961 PMC4987924

[34] Xie Z, Bailey A, Kuleshov MV, Clarke DJB, Evangelista JE, Jenkins SL, et al. Gene set knowledge discovery with Enrichr. Curr Protoc. 2021;1(3):e90. doi: 10.1002/cpz1.90 33780170 PMC8152575

[35] Kus ME, Sahin C, Kilic E, Askin A, Ozgur MM, Karahanogullari G, et al. A novel visual analysis interface enables integrative analyses of cancer transcriptomics data and iden-tifies potential markers of immunotherapy response via machine learning. 2023. doi: 10.1101/2023.08.14.553075

[36] Szklarczyk D, Franceschini A, Wyder S, Forslund K, Heller D, Huerta-Cepas J, et al. STRING v10: protein-protein interaction networks, integrated over the tree of life. Nucleic Acids Res. 2015;43(Database issue):D447-52. doi: 10.1093/nar/gku1003 25352553 PMC4383874

[37] Zuo Y, He Z, Chen Y, Dai L, et al. Dual role of ANGPTL4 in inflammation. Inflamm Res. 2023;72(6):1303–13. doi: 10.1007/s00011-023-01753-9 37300585 PMC10256975

[38] Hübers C, Abdul Pari AA, Grieshober D, Petkov M, Schmidt A, Messmer T, et al. Primary tumor-derived systemic nANGPTL4 inhibits metastasis. J Exp Med. 2023;220(1):e20202595. doi: 10.1084/jem.20202595 36269299 PMC9595206

[39] Tan MJ, Teo Z, Sng MK, Zhu P, Tan NS. Emerging roles of angiopoietin-like 4 in human cancer. Mol Cancer Res. 2012;10(6):677–88. doi: 10.1158/1541-7786.MCR-11-0519 22661548

[40] Tanaka T, Imamura T, Irie A, Yoneda M, Imamura R, Kikuchi K, et al. Association of high cellular expression and plasma concentration of angiopoietin-like 4 with tongue cancer lung metastasis and poor prognosis. Oncol Lett. 2022;24(3):299. doi: 10.3892/ol.2022.13419 35949602 PMC9353233

[41] Tanaka J, Irié T, Yamamoto G, Yasuhara R, Isobe T, Hokazono C, et al. ANGPTL4 regulates the metastatic potential of oral squamous cell carcinoma. J Oral Pathol Med. 2015;44(2):126–33. doi: 10.1111/jop.12212 25060575

[42] Jones D, Pereira ER, Padera TP. Growth and immune evasion of lymph node metastasis. Front Oncol. 2018;8:36. doi: 10.3389/fonc.2018.00036 29527513 PMC5829610

[43] Padua D, Zhang XH-F, Wang Q, Nadal C, Gerald WL, Gomis RR, et al. TGFbeta primes breast tumors for lung metastasis seeding through angiopoietin-like 4. Cell. 2008;133(1):66–77. doi: 10.1016/j.cell.2008.01.046 18394990 PMC2390892

[44] Li H, Ge C, Zhao F, Yan M, Hu C, Jia D, et al. Hypoxia-inducible factor 1 alpha-activated angiopoietin-like protein 4 contributes to tumor metastasis via vascular cell adhesion molecule-1/integrin β1 signaling in human hepatocellular carcinoma. Hepatology. 2011;54(3):910–9. doi: 10.1002/hep.24479 21674552

[45] Nakayama T, Hirakawa H, Shibata K, Nazneen A, Abe K, Nagayasu T, et al. Expression of angiopoietin-like 4 (ANGPTL4) in human colorectal cancer: ANGPTL4 promotes venous invasion and distant metastasis. Oncol Rep. 2011;25(4):929–35. doi: 10.3892/or.2011.1176 21308352

[46] Fan X, Li B, Zhang F, Liu M, Kwan H-Y, Liu Z, et al. FGF19-activated hepatic stellate cells release ANGPTL4 that promotes colorectal cancer liver metastasis. Adv Sci (Weinh). 2025;12(7):e2413525. doi: 10.1002/advs.202413525 39716892 PMC11831508

[47] Avalle L, Raggi L, Monteleone E, Savino A, Viavattene D, Statello L, et al. STAT3 induces breast cancer growth via ANGPTL4, MMP13 and STC1 secretion by cancer associated fibroblasts. Oncogene. 2022;41(10):1456–67. doi: 10.1038/s41388-021-02172-y 35042959

[48] XiongZ, ZhuangR-L, YuS-L, XieZ-X, PengS-R, LiZ-A, et al. Cancer-associated fibroblasts regulate mitochondrial metabolism and inhibit chemosensitivity via ANGPTL4-IQGAP1 axis in prostate cancer. J Adv Res. 2024:S2090-1232(24)00559-9. doi: 10.1016/j.jare.2024.12.003 39647634

[49] Bajwa P, Kordylewicz K, Bilecz A, Lastra RR, Wroblewski K, Rinkevich Y, et al. Cancer-associated mesothelial cell-derived ANGPTL4 and STC1 promote the early steps of ovarian cancer metastasis. JCI Insight. 2023;8(6):e163019. doi: 10.1172/jci.insight.163019 36795484 PMC10070116

[50] Rankin EB, Giaccia AJ. Hypoxic control of metastasis. Science. 2016;352(6282):175–80. doi: 10.1126/science.aaf4405 27124451 PMC4898055

[51] Chen Z, Han F, Du Y, Shi H, Zhou W. Hypoxic microenvironment in cancer: molecular mechanisms and therapeutic interventions. Signal Transduct Target Ther. 2023;8(1):70. doi: 10.1038/s41392-023-01332-8 36797231 PMC9935926

[52] Baba K, Kitajima Y, Miyake S, Nakamura J, Wakiyama K, Sato H, et al. Hypoxia-induced ANGPTL4 sustains tumour growth and anoikis resistance through different mechanisms in scirrhous gastric cancer cell lines. Sci Rep. 2017;7(1):11127. doi: 10.1038/s41598-017-11769-x 28894280 PMC5594024

[53] Golijanin B, Malshy K, Khaleel S, Lagos G, Amin A, Cheng L, et al. Evolution of the HIF targeted therapy in clear cell renal cell carcinoma. Cancer Treat Rev. 2023;121:102645. doi: 10.1016/j.ctrv.2023.102645 37879247

[54] Jin Z, De U, Tithi TI, Kleberg J, Nataraj A, Jolley E, et al. ANGPTL4 suppresses clear cell renal cell carcinoma via inhibition of lysosomal acid lipase. Cancer Res Commun. 2024;4(8):2242–54. doi: 10.1158/2767-9764.CRC-24-0016 39105498 PMC11348483

[55] Izraely S, Ben-Menachem S, Sagi-Assif O, Meshel T, Marzese DM, Ohe S, et al. ANGPTL4 promotes the progression of cutaneous melanoma to brain metastasis. Oncotarget. 2017;8(44):75778–96. doi: 10.18632/oncotarget.19018 29100268 PMC5652662

[56] Shih C-Y, Cheng Y-C, Hsieh C, Tseng T, Jiang S, Lee S-C. Drug-selected population in melanoma A2058 cells as melanoma stem-like cells retained angiogenic features - the potential roles of heparan-sulfate binding ANGPTL4 protein. Aging (Albany NY). 2020;12(22):22700–18. doi: 10.18632/aging.103890 33196458 PMC7746371

[57] Fernández-Hernando C, Suárez Y. ANGPTL4: a multifunctional protein involved in metabolism and vascular homeostasis. Curr Opin Hematol. 2020;27(3):206–13. doi: 10.1097/MOH.0000000000000580 32205586 PMC9013473

[58] Xu J, Wu F, Zhu Y, Wu T, Cao T, Gao W, et al. ANGPTL4 regulates ovarian cancer progression by activating the ERK1/2 pathway. Cancer Cell Int. 2024;24(1):54. doi: 10.1186/s12935-024-03246-z 38311733 PMC10838463

[59] Liao Z, Lim JJH, Lee JXT, Chua D, Vos MIG, Yip YS, et al. Attenuating epithelial-to-mesenchymal transition in cancer through angiopoietin-like 4 inhibition in a 3D tumor microenvironment model. Adv Healthc Mater. 2024;13(10):e2303481. doi: 10.1002/adhm.202303481 37987244

[60] Zhou S, Wang R, Xiao H. Adipocytes induce the resistance of ovarian cancer to carboplatin through ANGPTL4. Oncol Rep. 2020;44(3):927–38. doi: 10.3892/or.2020.7647 32705217 PMC7388553

[61] Kolb R, Kluz P, Tan ZW, Borcherding N, Bormann N, Vishwakarma A, et al. Obesity-associated inflammation promotes angiogenesis and breast cancer via angiopoietin-like 4. Oncogene. 2019;38(13):2351–63. doi: 10.1038/s41388-018-0592-6 30518876 PMC6440811

[62] Li C, Wang Q, Luo Y, Xiang J. TAZ regulates the cisplatin resistance of epithelial ovarian cancer cells via the ANGPTL4/SOX2 axis. Anal Cell Pathol (Amst). 2022;2022:5632164. doi: 10.1155/2022/5632164 36247876 PMC9553699

[63] Wang D, Zhao C, Xu F, Zhang A, Jin M, Zhang K, et al. Cisplatin-resistant NSCLC cells induced by hypoxia transmit resistance to sensitive cells through exosomal PKM2. Theranostics. 2021;11(6):2860–75. doi: 10.7150/thno.51797 33456577 PMC7806469

[64] Wang Z, Han B, Zhang Z, Pan J, Xia H. Expression of angiopoietin-like 4 and tenascin C but not cathepsin C mRNA predicts prognosis of oral tongue squamous cell carcinoma. Biomarkers. 2010;15(1):39–46. doi: 10.3109/13547500903261362 19775228

[65] Nakayama T, Hirakawa H, Shibata K, Abe K, Nagayasu T, Taguchi T. Expression of angiopoietin-like 4 in human gastric cancer: ANGPTL4 promotes venous invasion. Oncol Rep. 2010;24(3):599–606. doi: 10.3892/or_00000897 20664963

[66] Shibata K, Nakayama T, Hirakawa H, Hidaka S, Nagayasu T. Clinicopathological significance of angiopoietin-like protein 4 expression in oesophageal squamous cell carcinoma. J Clin Pathol. 2010;63(12):1054–8. doi: 10.1136/jcp.2010.078600 20861003

[67] Li H, Ge C, Zhao F, Yan M, Hu C, Jia D, et al. Hypoxia-inducible factor 1 alpha-activated angiopoietin-like protein 4 contributes to tumor metastasis via vascular cell adhesion molecule-1/integrin β1 signaling in human hepatocellular carcinoma. Hepatology. 2011;54(3):910–9. doi: 10.1002/hep.24479 21674552

[68] Mannelqvist M, Stefansson IM, Bredholt G, Hellem Bø T, Oyan AM, Jonassen I, et al. Gene expression patterns related to vascular invasion and aggressive features in endometrial cancer. Am J Pathol. 2011;178(2):861–71. doi: 10.1016/j.ajpath.2010.10.040 21281818 PMC3070569

[69] Akishima-Fukasawa Y, Ishikawa Y, Akasaka Y, Uzuki M, Inomata N, Yokoo T, et al. Histopathological predictors of regional lymph node metastasis at the invasive front in early colorectal cancer. Histopathology. 2011;59(3):470–81. doi: 10.1111/j.1365-2559.2011.03964.x 22034887

[70] Yi J, Pan B, Xiong L, Song H. Clinical significance of angiopoietin-like protein 4 expression in tissue and serum of esophageal squamous cell carcinoma patients. Med Oncol. 2013;30(3):680. doi: 10.1007/s12032-013-0680-y 23925665 PMC3755218

[71] Ng KT-P, Xu A, Cheng Q, Guo DY, Lim ZX-H, Sun CK-W, et al. Clinical relevance and therapeutic potential of angiopoietin-like protein 4 in hepatocellular carcinoma. Mol Cancer. 2014;13:196. doi: 10.1186/1476-4598-13-196 25148701 PMC4149052

[72] Shafik NM, Mohamed DA, Bedder AE, El-Gendy AM. Significance of tissue expression and serum levels of angiopoietin-like protein 4 in breast cancer progression: link to NF-κB /P65 activity and pro-inflammatory cytokines. Asian Pac J Cancer Prev. 2015;16(18):8579–87. doi: 10.7314/apjcp.2015.16.18.8579 26745120

[73] Tanaka J, Irié T, Yamamoto G, Yasuhara R, Isobe T, Hokazono C, et al. ANGPTL4 regulates the metastatic potential of oral squamous cell carcinoma. J Oral Pathol Med. 2015;44(2):126–33. doi: 10.1111/jop.12212 25060575

[74] Li X, Chen T, Shi Q, Li J, Cai S, Zhou P, et al. Angiopoietin-like 4 enhances metastasis and inhibits apoptosis via inducing bone morphogenetic protein 7 in colorectal cancer cells. Biochem Biophys Res Commun. 2015;467(1):128–34. doi: 10.1016/j.bbrc.2015.09.104 26417691

[75] Kubo H, Kitajima Y, Kai K, Nakamura J, Miyake S, Yanagihara K, et al. Regulation and clinical significance of the hypoxia-induced expression of ANGPTL4 in gastric cancer. Oncol Lett. 2016;11(2):1026–34. doi: 10.3892/ol.2015.4011 26893686 PMC4734226

[76] Zhu X, Guo X, Wu S, Wei L. ANGPTL4 correlates with NSCLC progression and regulates epithelial-mesenchymal transition via ERK pathway. Lung. 2016;194(4):637–46. doi: 10.1007/s00408-016-9895-y 27166634

[77] Huang Z, Xie J, Lin S, Li S, Huang Z, Wang Y, et al. The downregulation of ANGPTL4 inhibits the migration and proliferation of tongue squamous cell carcinoma. Arch Oral Biol. 2016;71:144–9. doi: 10.1016/j.archoralbio.2016.07.011 27505034

[78] Hata S, Nomura T, Iwasaki K, Sato R, Yamasaki M, Sato F, et al. Hypoxia-induced angiopoietin-like protein 4 as a clinical biomarker and treatment target for human prostate cancer. Oncol Rep. 2017;38(1):120–8. doi: 10.3892/or.2017.5669 28560449

[79] Aung TM, Silsirivanit A, Jusakul A, Chan-On W, Proungvitaya T, Roytrakul S, et al. Prediction of angiopoietin-like protein 4-related signaling pathways in cholangiocarcinoma cells. Cancer Genomics Proteomics. 2022;19(4):490–502. doi: 10.21873/cgp.20335 35732325 PMC9247875

[80] Kamaludin Z, Siddig A, Yaacob NM, Lam AK, Rahman WFWA. Angiopoietin-like protein 4 and insulin-like growth factor-1 expression in invasive breast carcinoma in young women. Pathophysiology. 2022;29(1):9–23. doi: 10.3390/pathophysiology29010002 35366286 PMC8955684

[81] Dong D, Jia L, Zhou Y, Ren L, Li J, Zhang J. Serum level of ANGPTL4 as a potential biomarker in renal cell carcinoma. Urol Oncol. 2017;35(5):279–85. doi: 10.1016/j.urolonc.2016.12.017 28110976

[82] Lee H-L, Chiou H-L, Wang S-S, Hung S-C, Chou M-C, Yang S-F, et al. WISP1 genetic variants as predictors of tumor development with urothelial cell carcinoma. Urol Oncol. 2018;36(4):160.e15-160.e21. doi: 10.1016/j.urolonc.2017.11.023 29277583

[83] Kirby MK, Ramaker RC, Gertz J, Davis NS, Johnston BE, Oliver PG, et al. RNA sequencing of pancreatic adenocarcinoma tumors yields novel expression patterns associated with long-term survival and reveals a role for ANGPTL4. Mol Oncol. 2016;10(8):1169–82. doi: 10.1016/j.molonc.2016.05.004 27282075 PMC5423196

[84] Dao T, Gapihan G, Leboeuf C, Hamdan D, Feugeas J-P, Boudabous H, et al. Expression of angiopoietin-like 4 fibrinogen-like domain (cANGPTL4) increases risk of brain metastases in women with breast cancer. Oncotarget. 2020;11(18):1590–602. doi: 10.18632/oncotarget.27553 32405335 PMC7210011

[85] Zheng X, Liu R, Zhou C, Yu H, Luo W, Zhu J, et al. ANGPTL4-mediated promotion of glycolysis facilitates the colonization of fusobacterium nucleatum in colorectal cancer. Cancer Res. 2021;81(24):6157–70. doi: 10.1158/0008-5472.CAN-21-2273 34645607 PMC9397643

[86] Mizuno S, Seishima R, Yamasaki J, Hattori K, Ogiri M, Matsui S, et al. Angiopoietin-like 4 promotes glucose metabolism by regulating glucose transporter expression in colorectal cancer. J Cancer Res Clin Oncol. 2022;148(6):1351–61. doi: 10.1007/s00432-022-03960-z 35195748 PMC11800850

[87] Yan HH, Jung KH, Lee JE, Son MK, Fang Z, Park JH, et al. ANGPTL4 accelerates KRASG12D-Induced acinar to ductal metaplasia and pancreatic carcinogenesis. Cancer Lett. 2021;519:185–98. doi: 10.1016/j.canlet.2021.07.036 34311032

