# Supplementary figures and images for: The role of ANGPTL4 in cancer: A meta-analysis of observational studies and multi-omics investigation
Source: PLoS One. 2025 Apr 15;20(4):e0320343. doi: 10.1371/journal.pone.0320343 (PMC11999138; doi:10.1371/journal.pone.0320343)

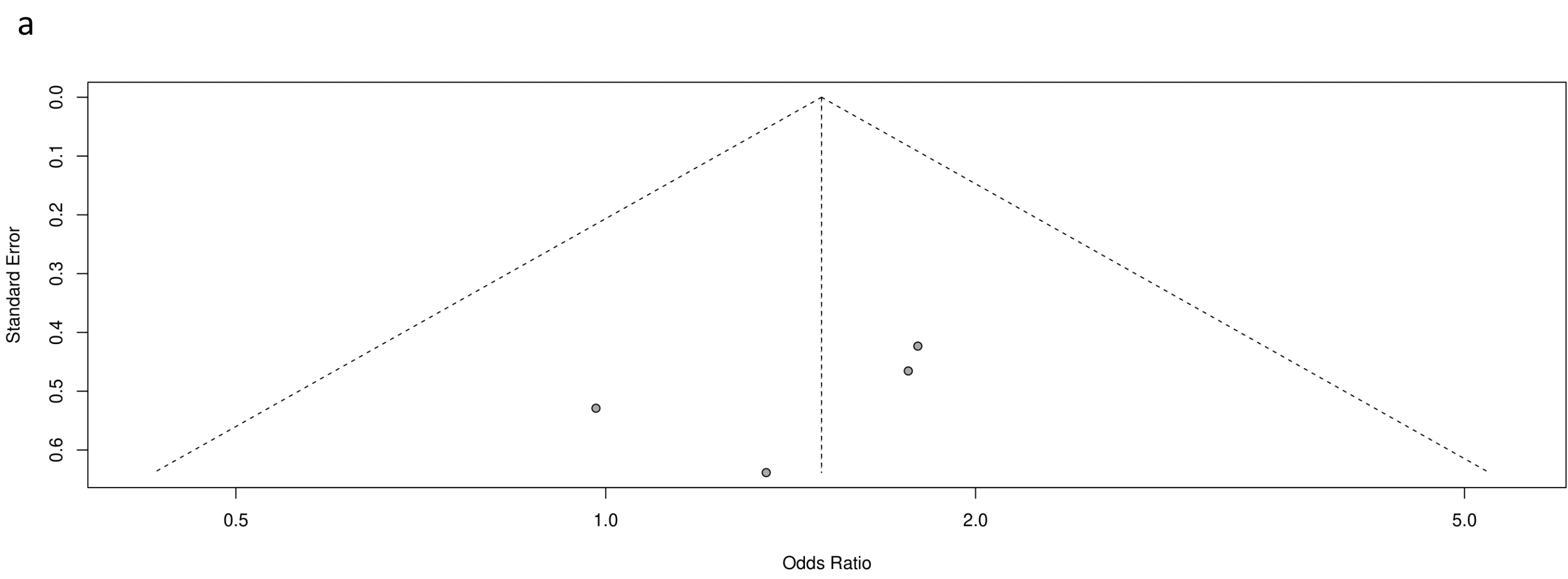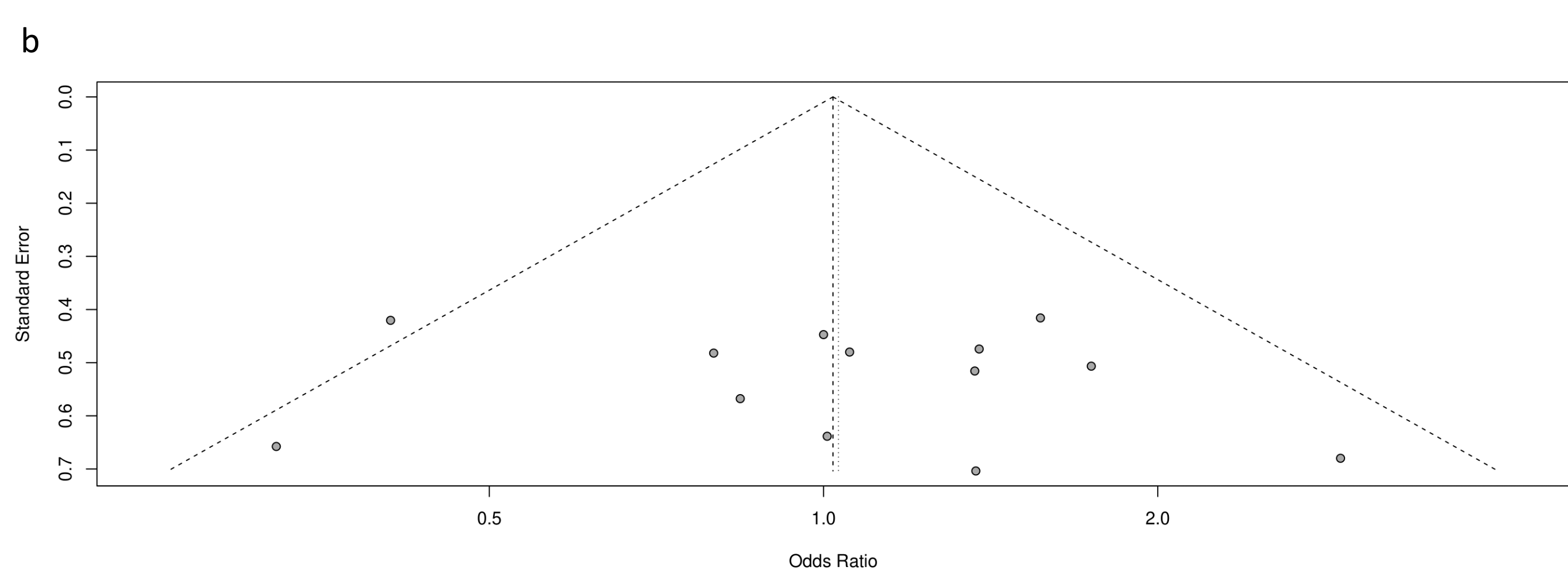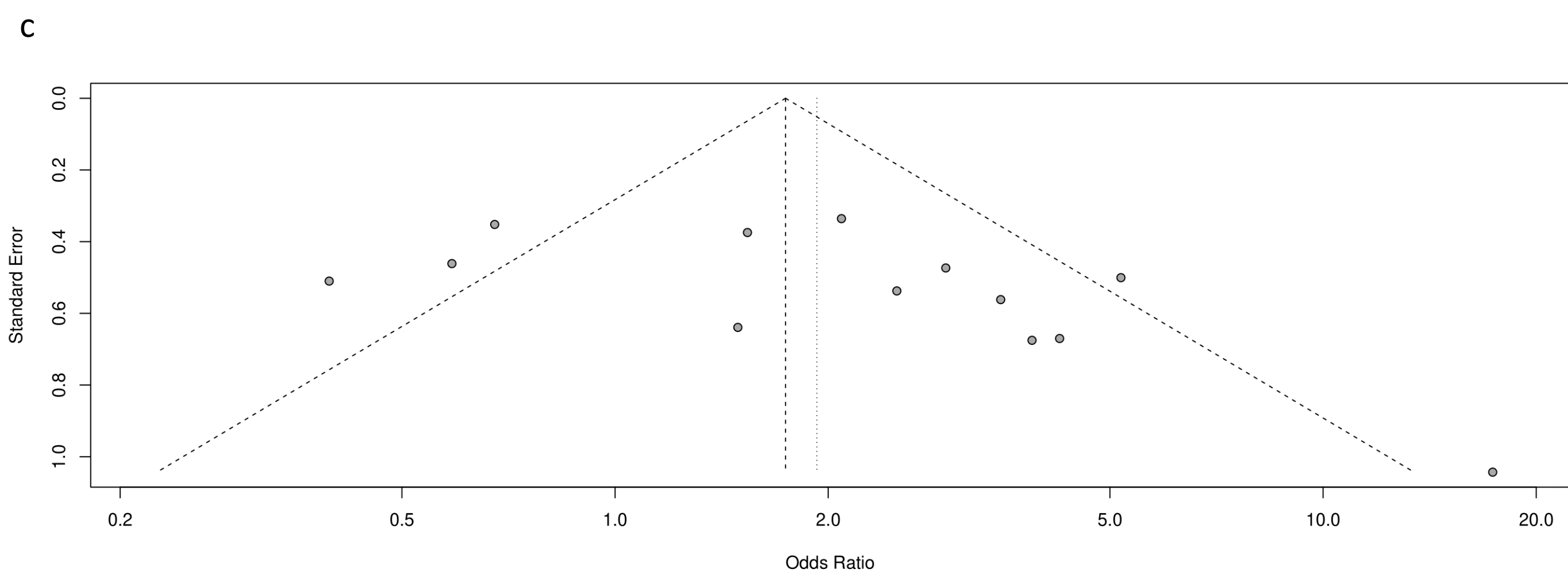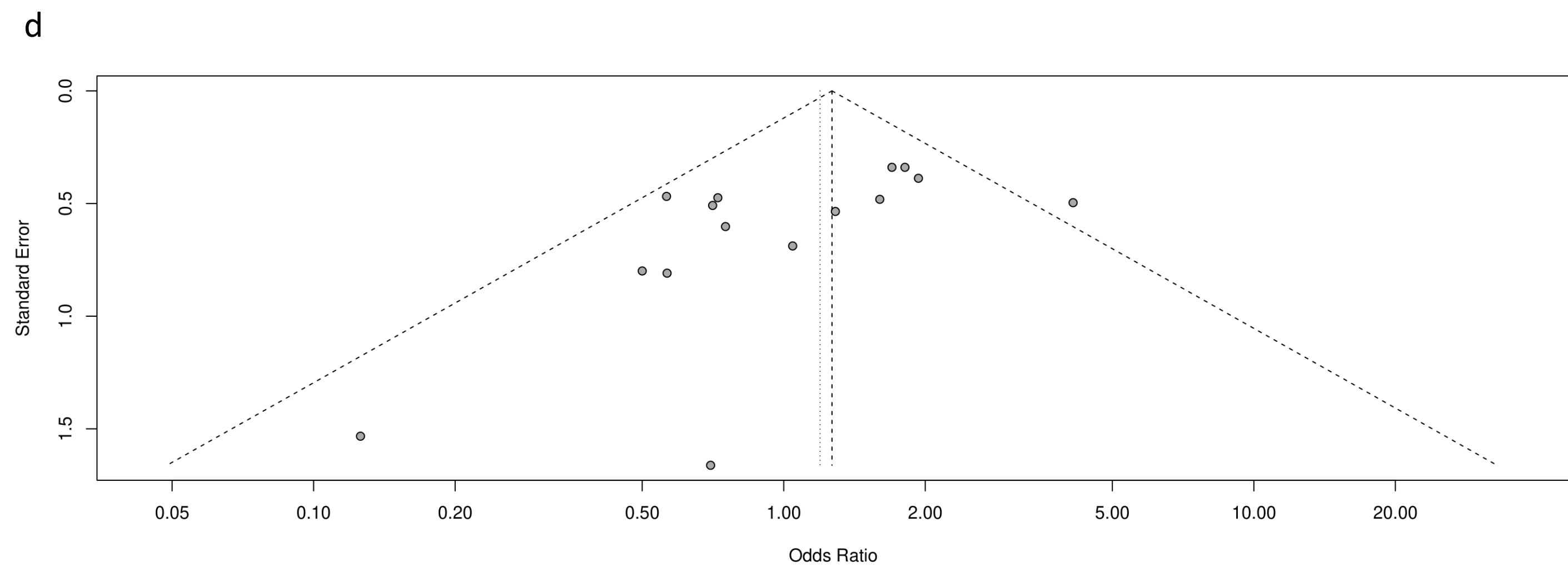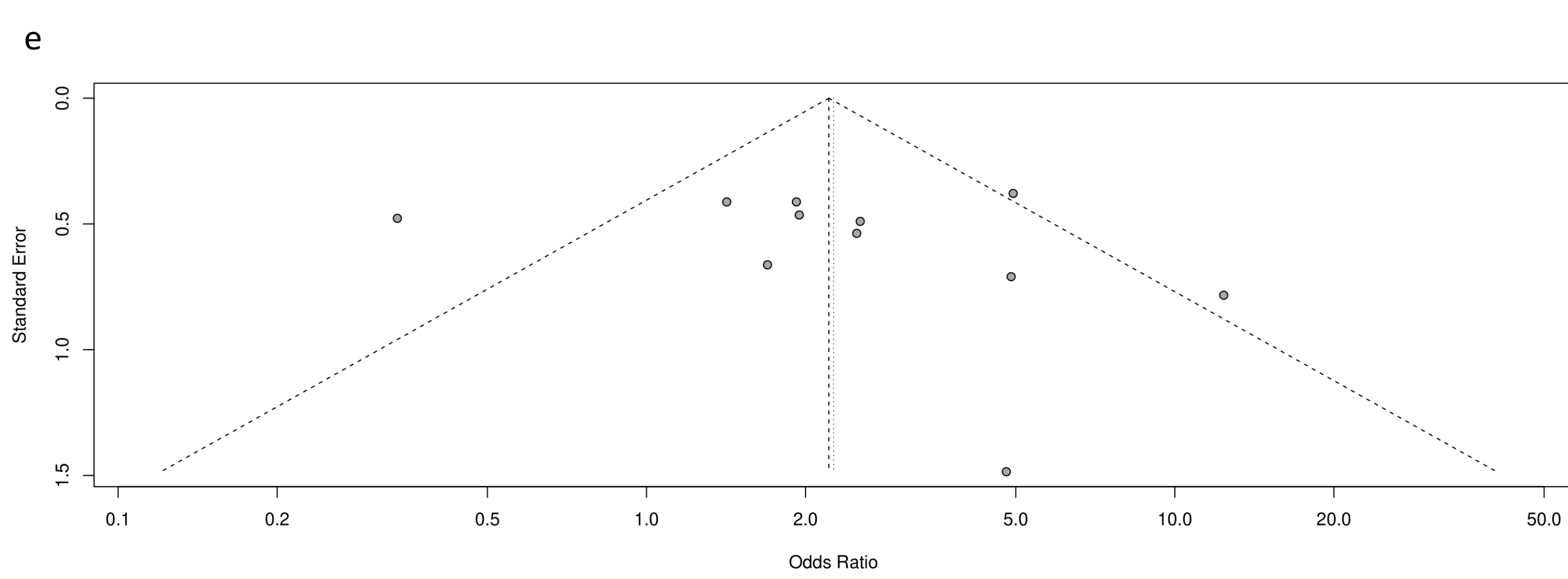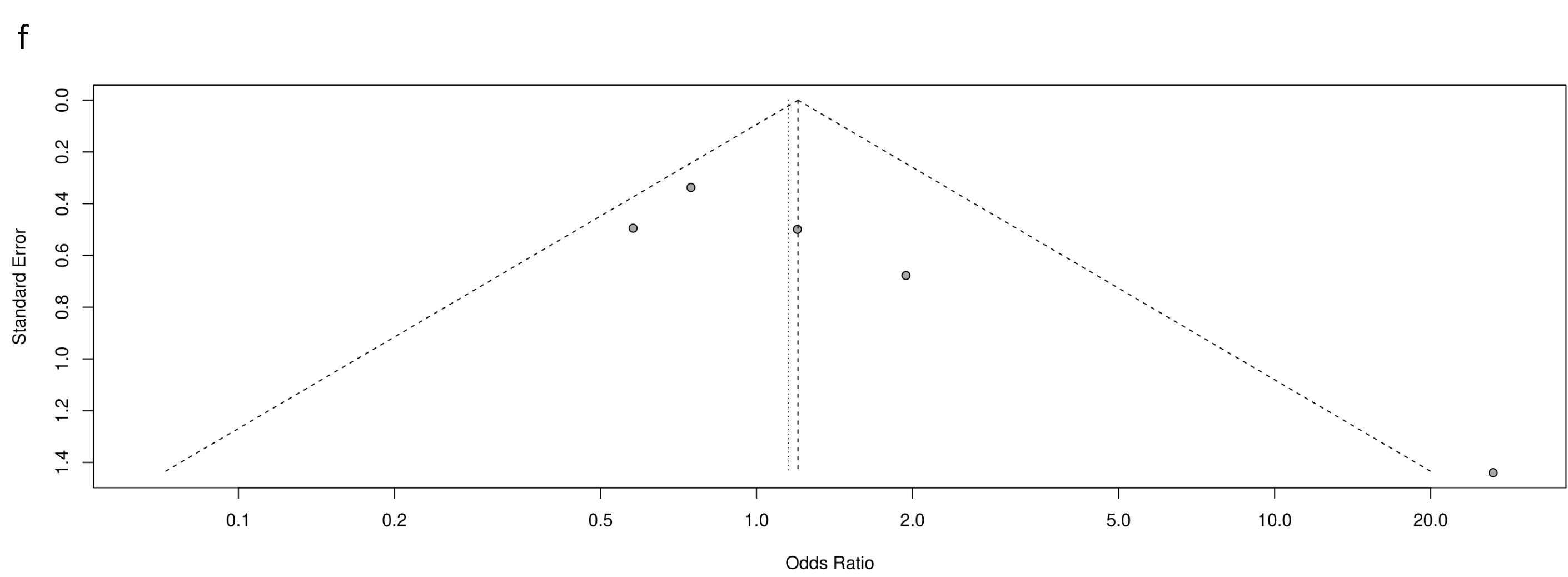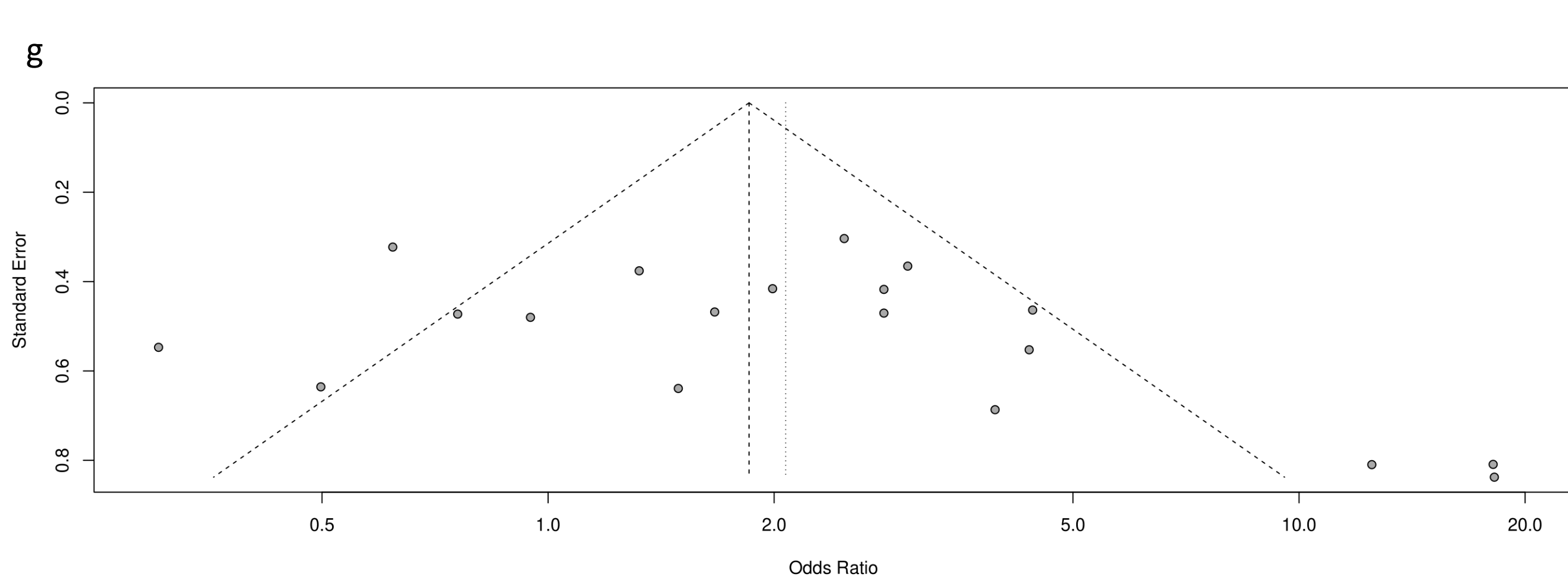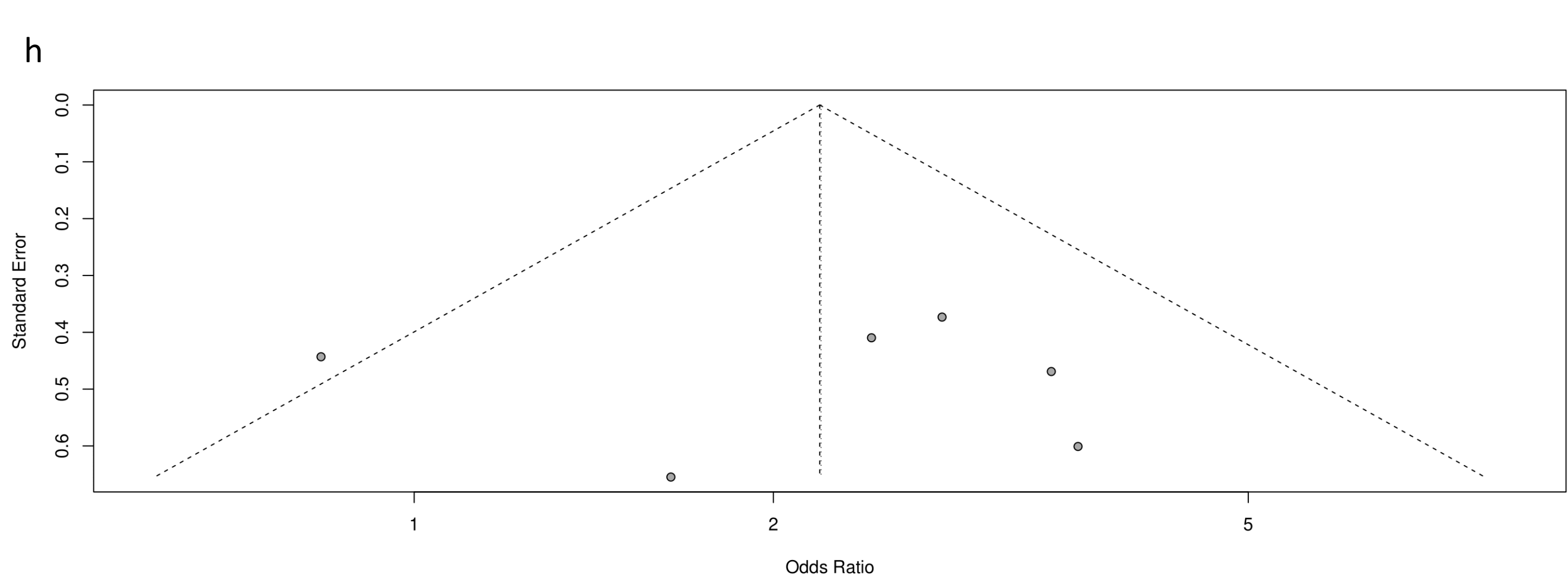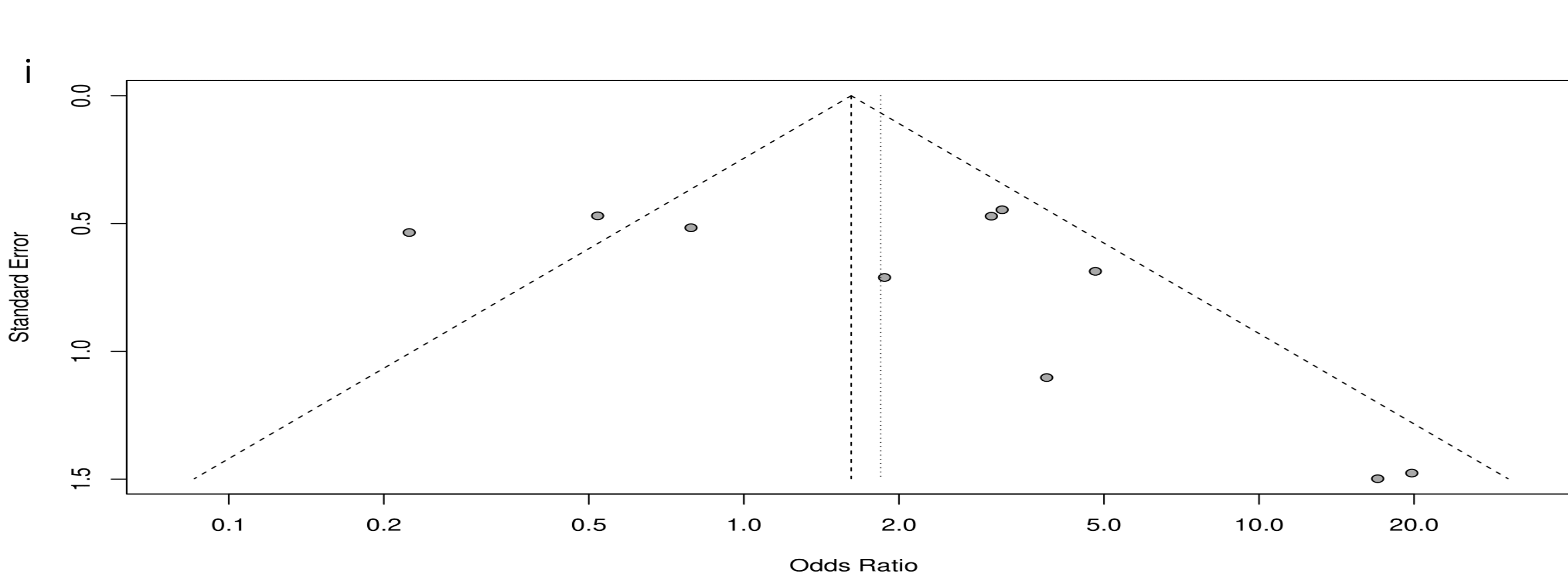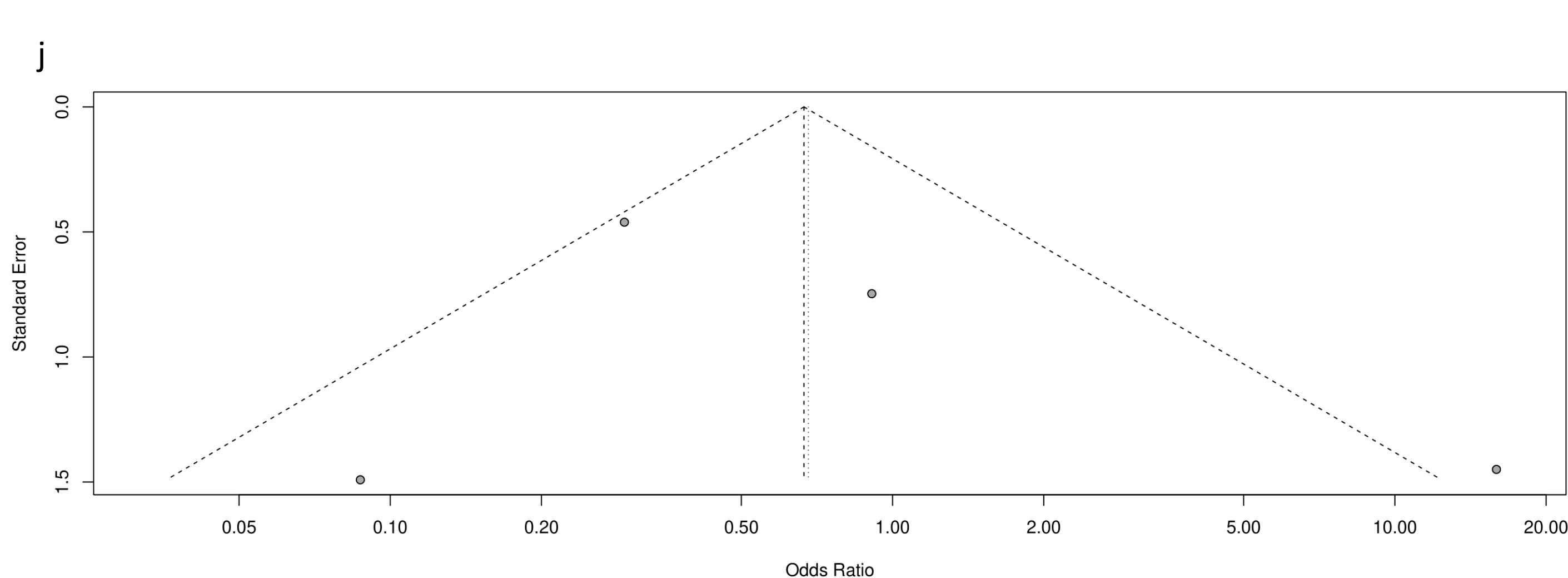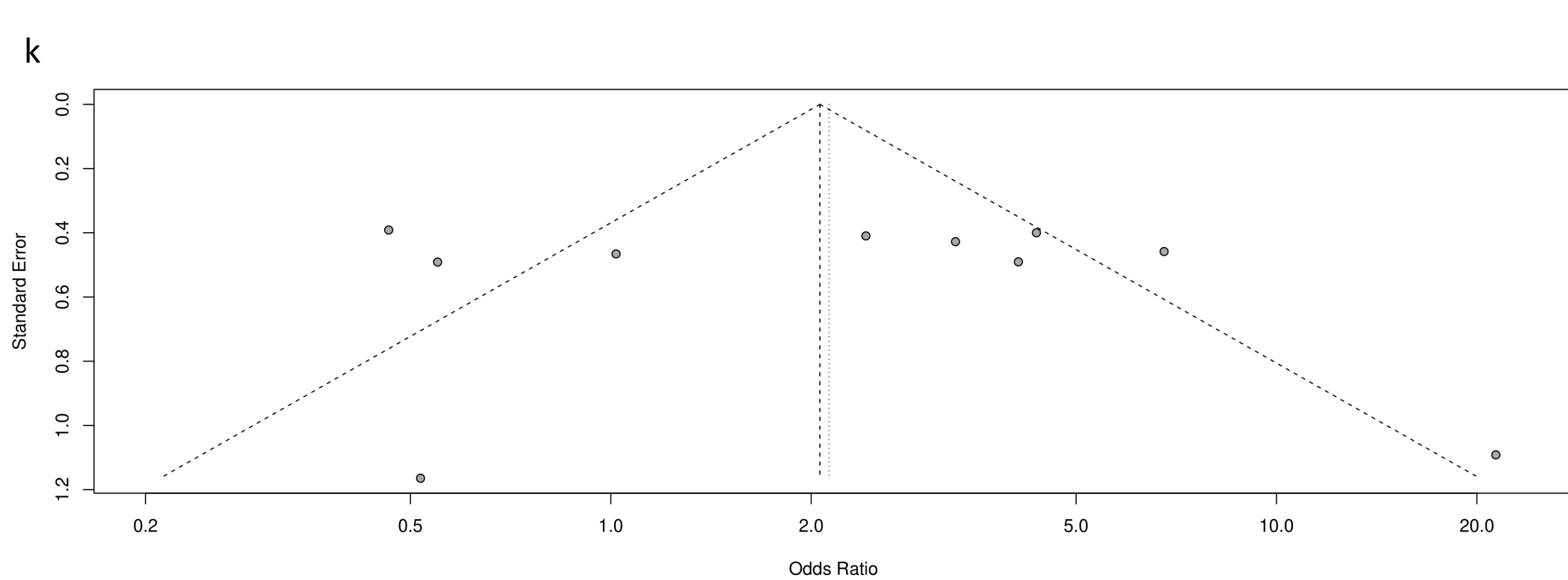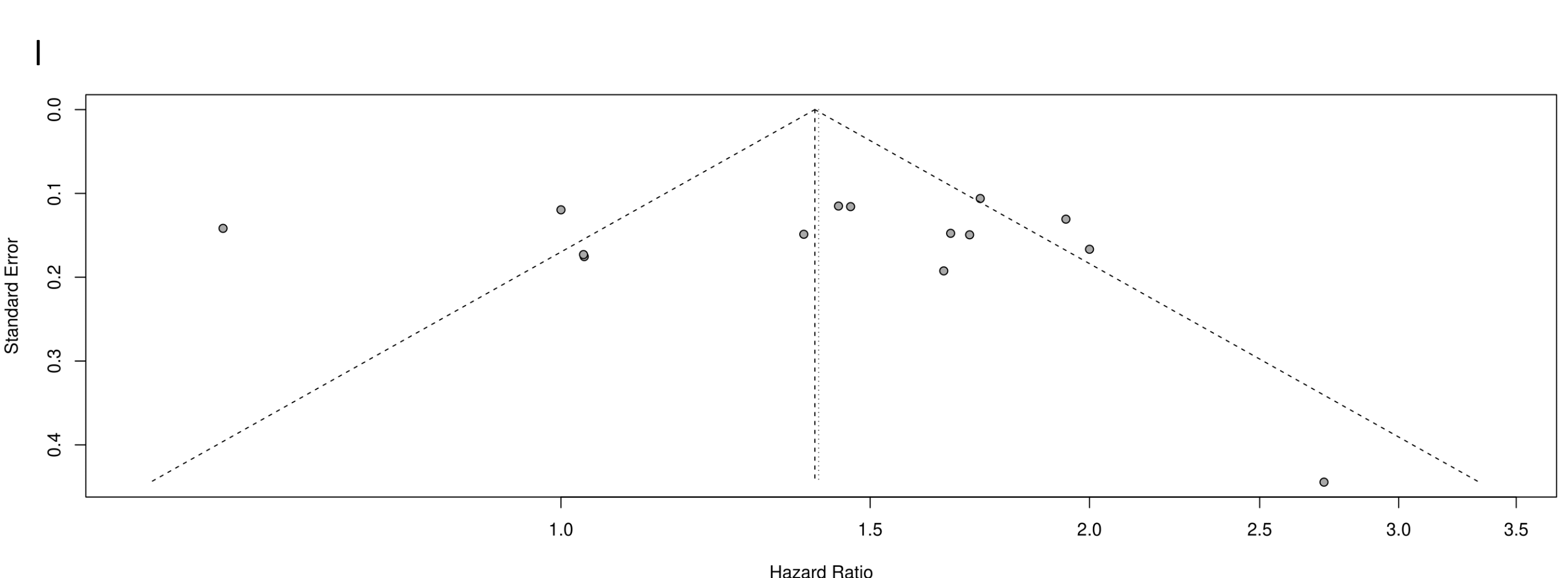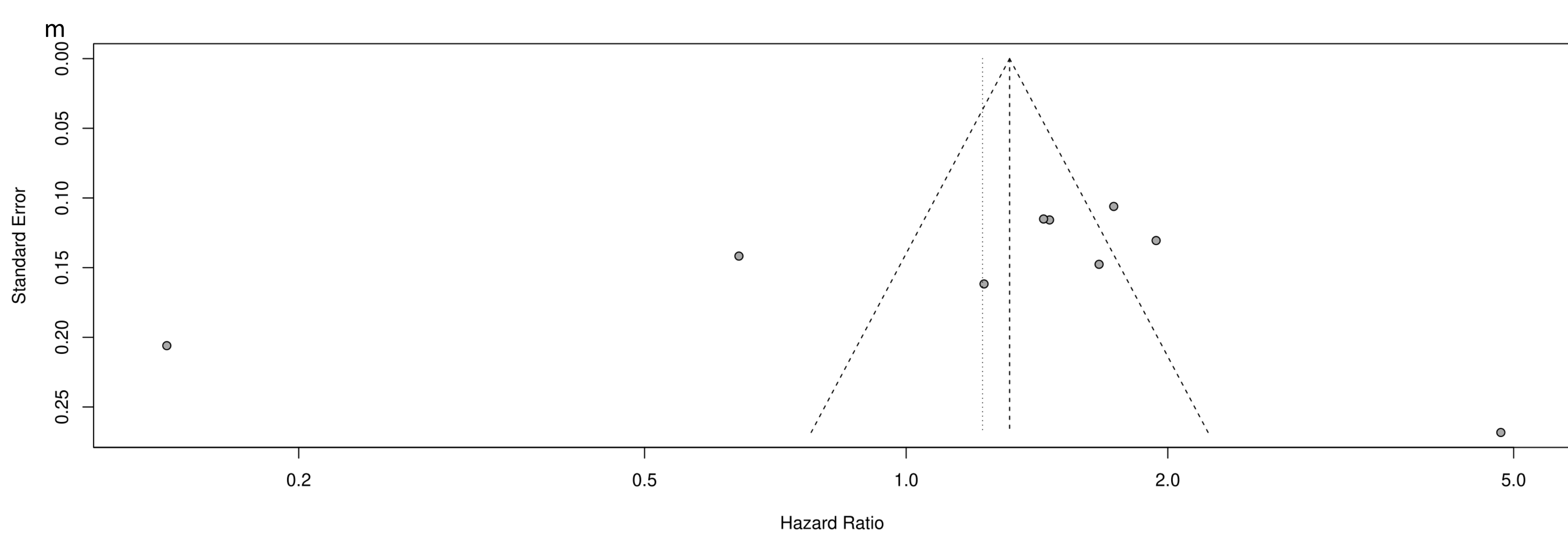

Supplement: S1 Fig — Figure S1 shows the funnel plots for all the pooled outcomes. (A) Age (B) Gender (C) TNM staging (D) Histological differentiation (E) T staging (F) Tumor size (G) Lymph node metastasis (H) Lymphatic Invasion (I) Distant metastasis (J) Local recurrence (K) Vascular invasion (L) Overall Survival (M) Disease-Free Survival. (PDF) [file pone.0320343.s001.pdf]

a

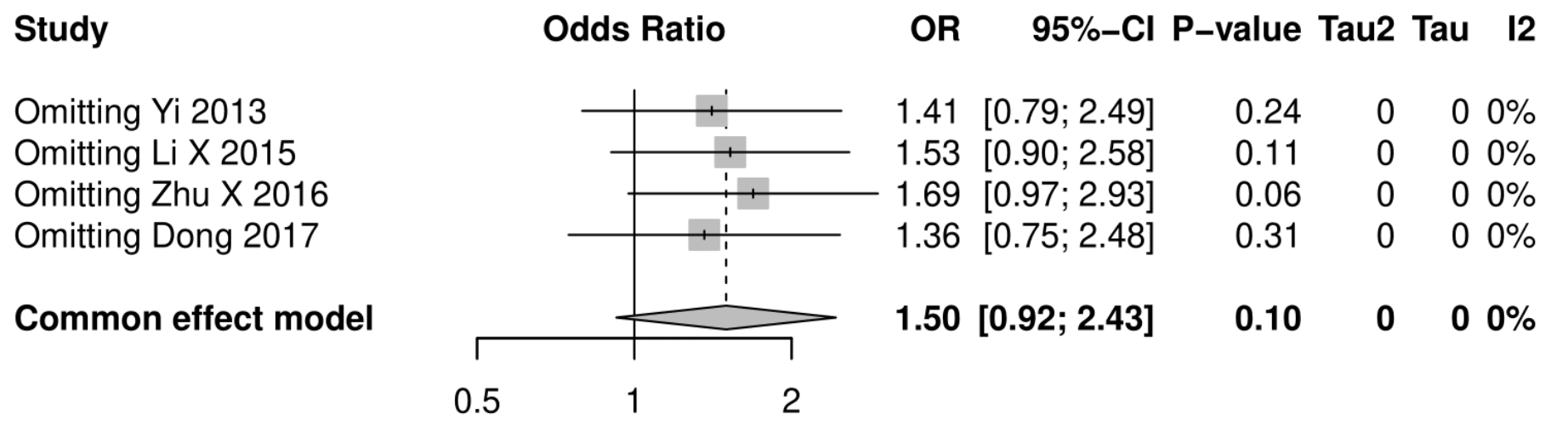

b

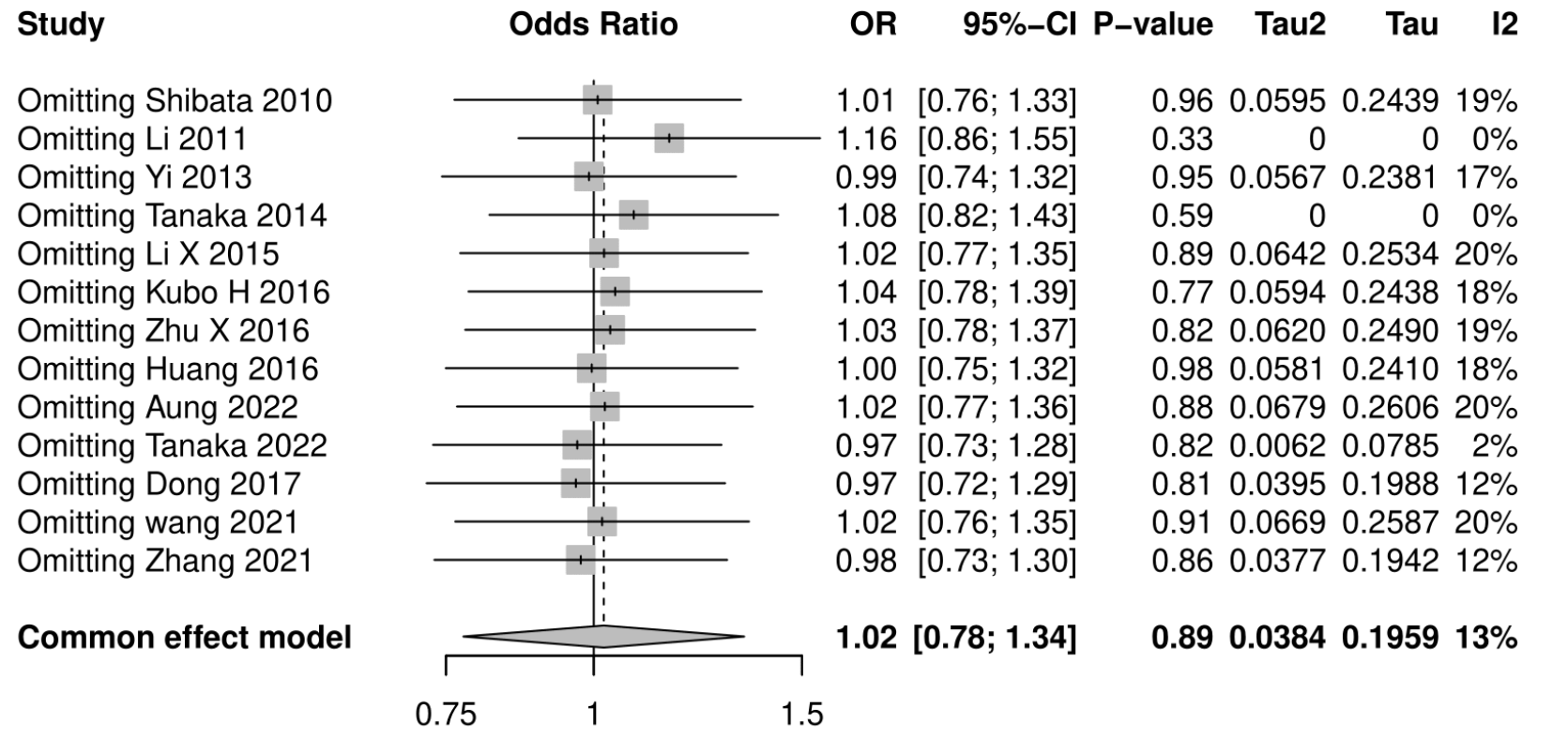

c

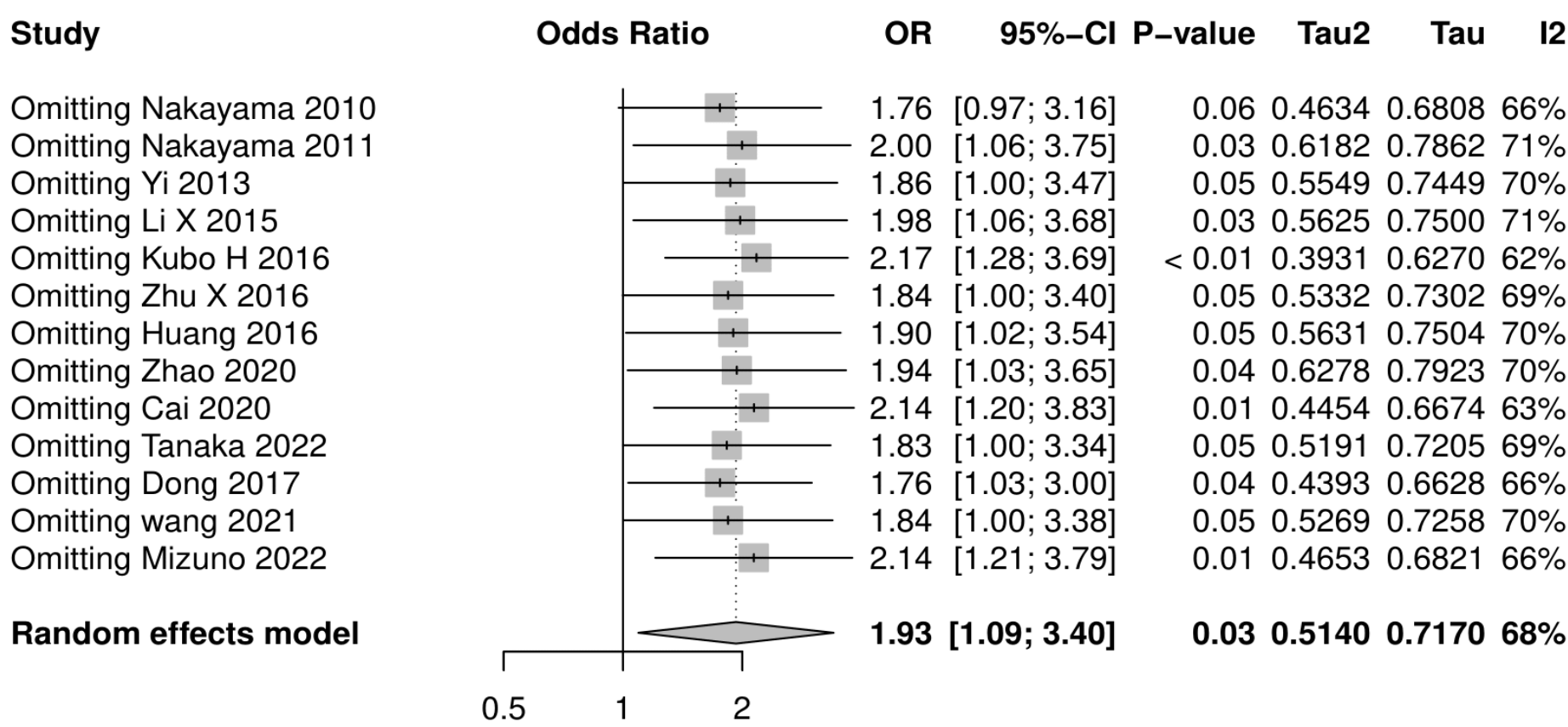

d

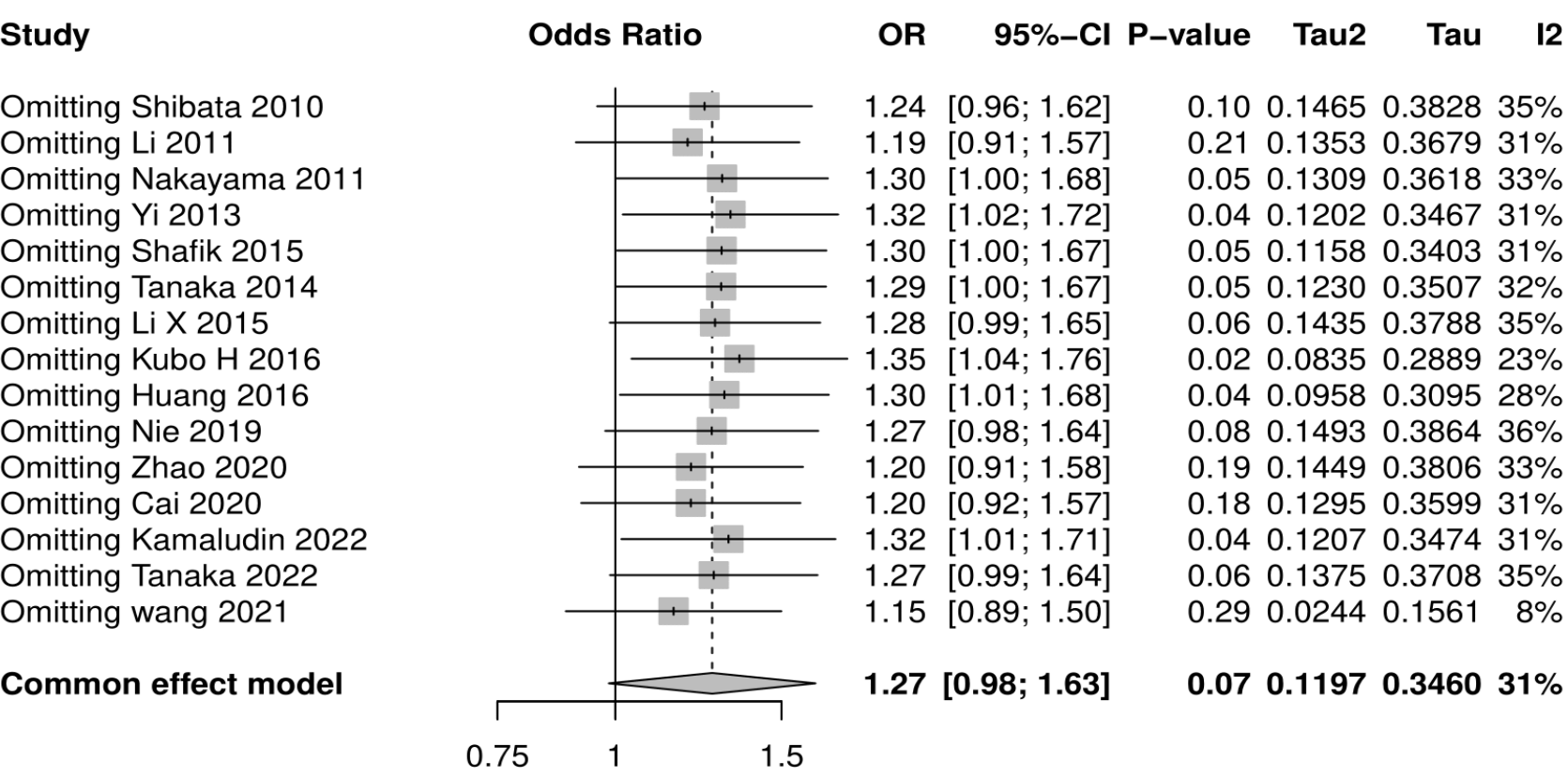

e

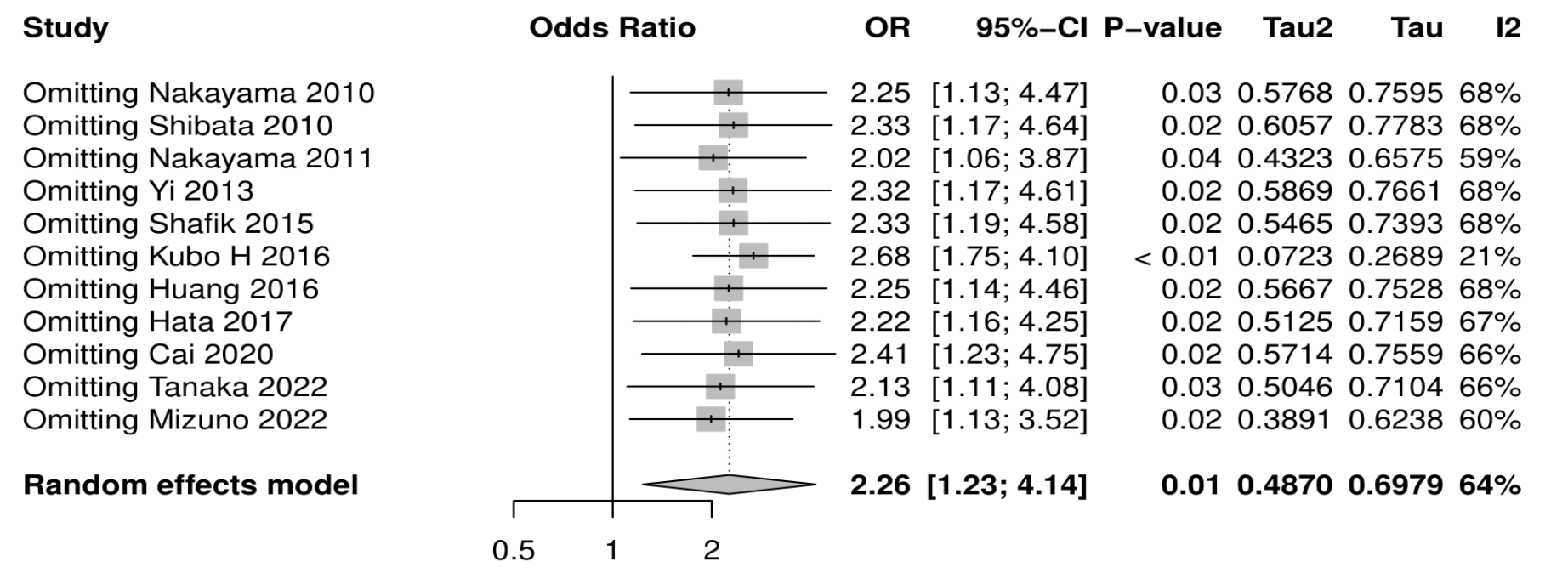

f

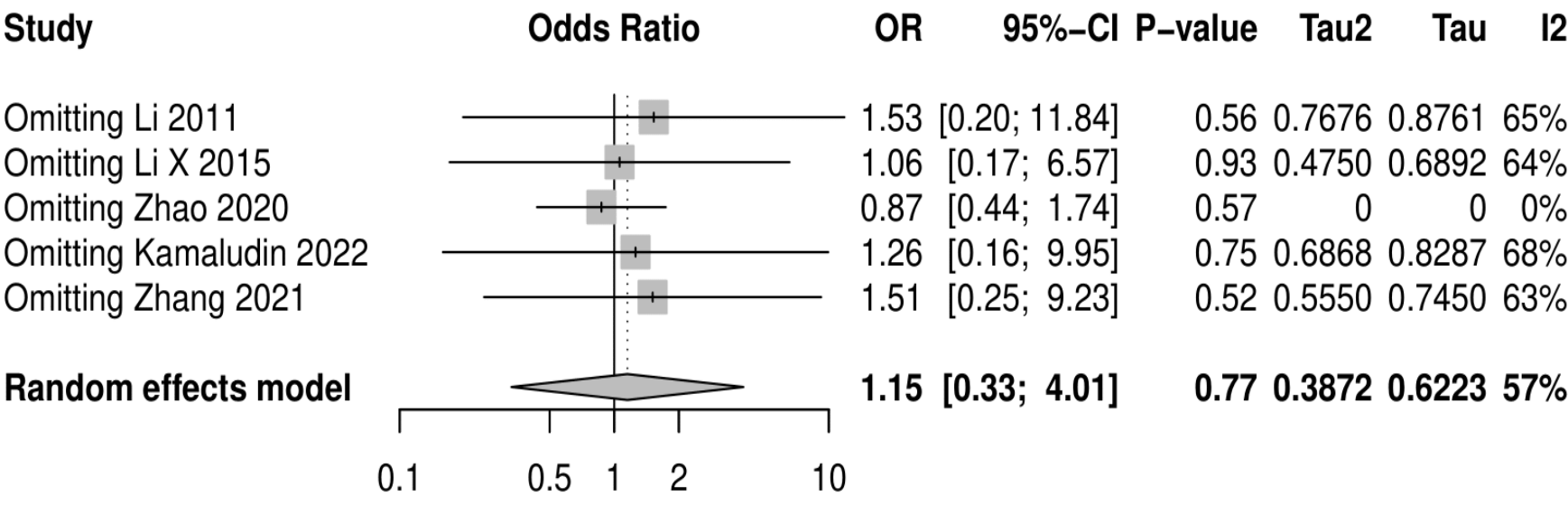

g

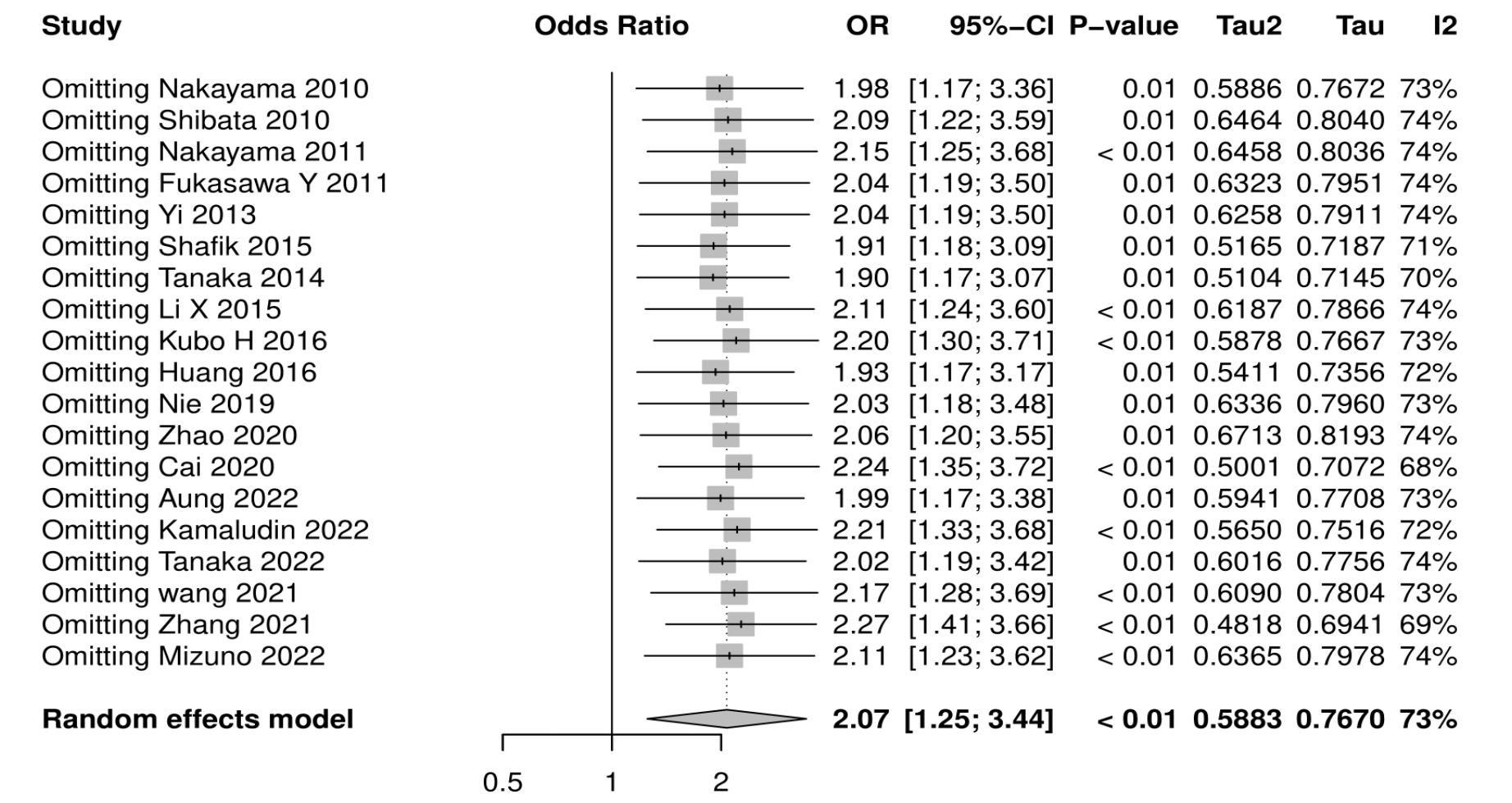

h

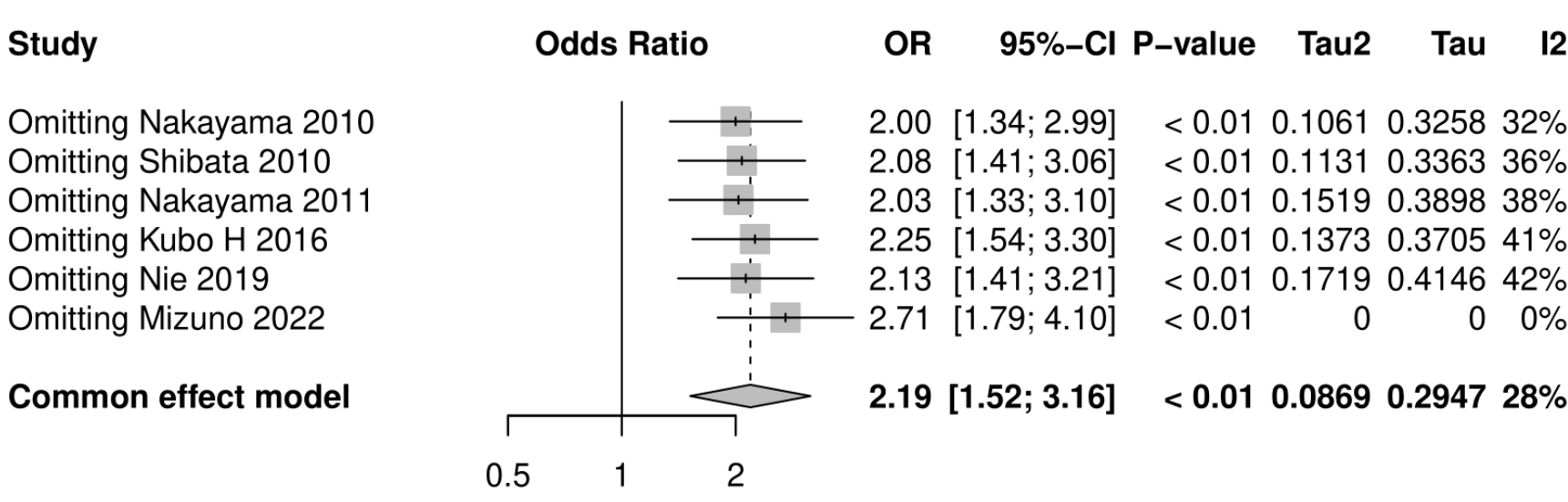

i

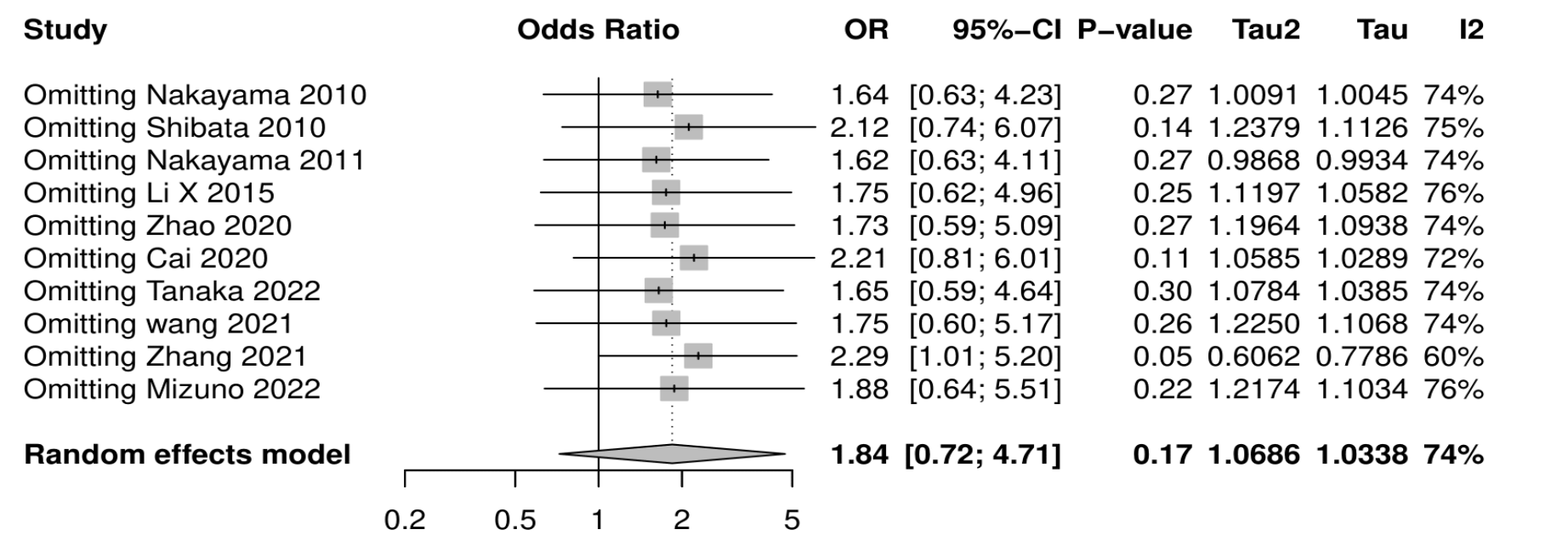

j

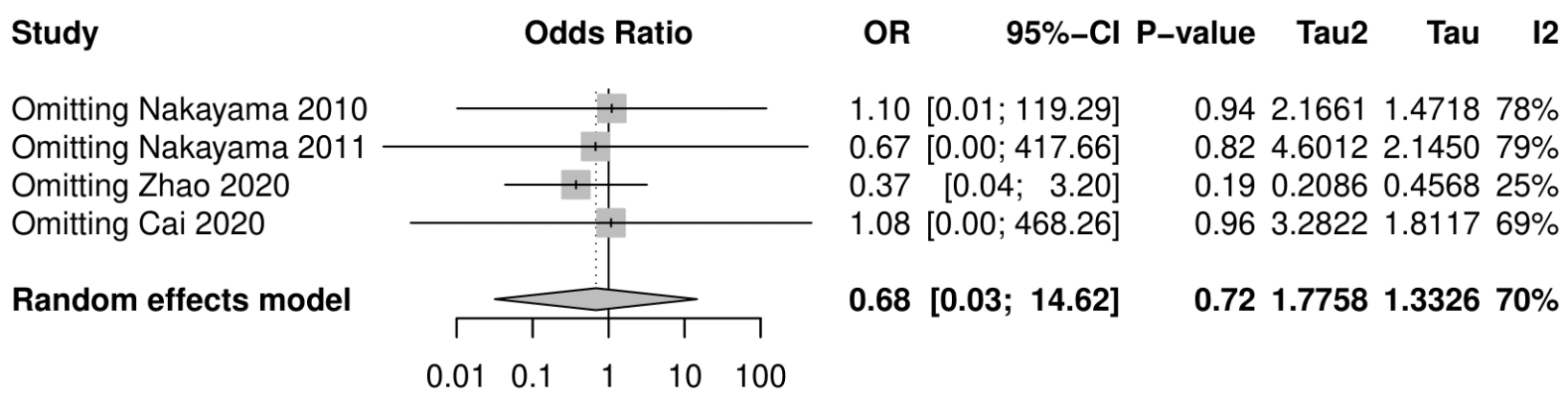

k

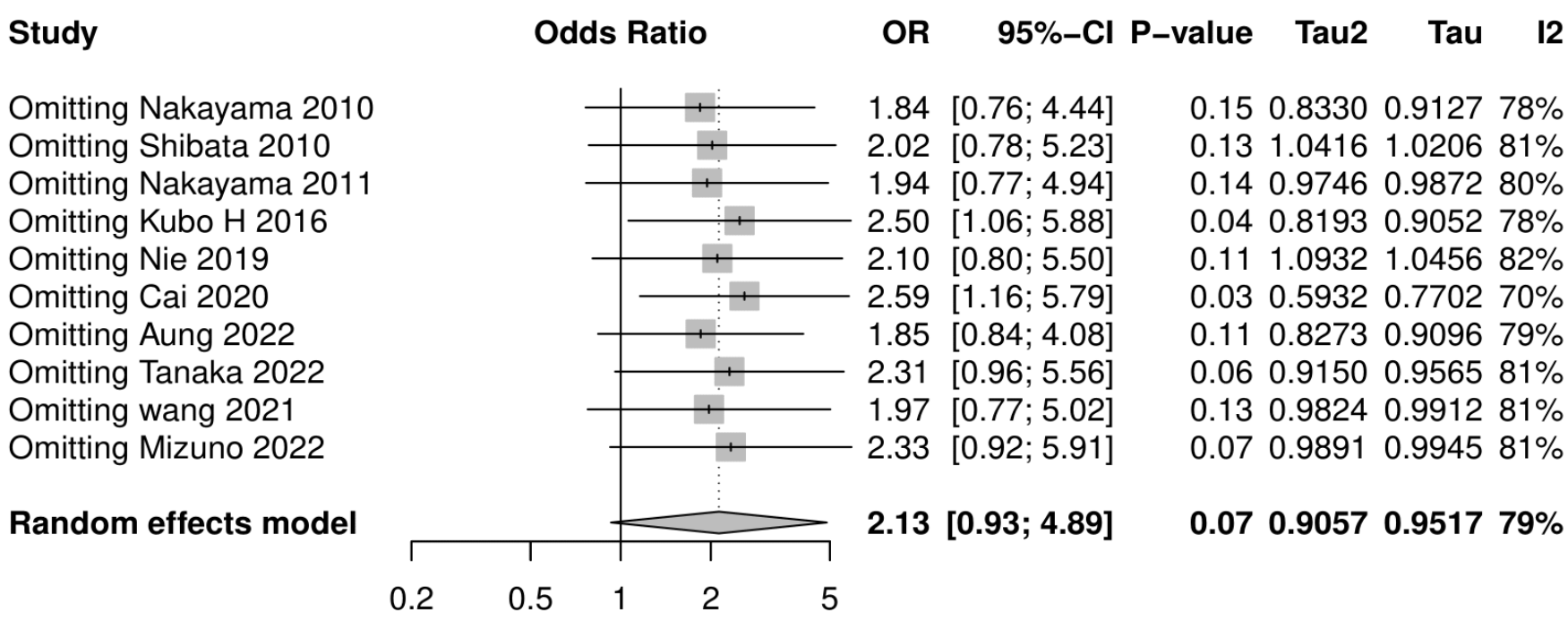

Supplement: S2 Fig — Figure S2 shows leave one out plots that represent the sensitivity analysis. (A) Age (B) Gender (C) TNM staging (D) Histological differentiation (E) T staging (F) Tumor size (G) Lymph node metastasis (H) Lymphatic Invasion (I) Distant metastasis (J) Local recurrence (K) Vascular invasion (L) Overall Survival (M) Disease-Free Survival. (PDF) [file pone.0320343.s002.pdf]

a

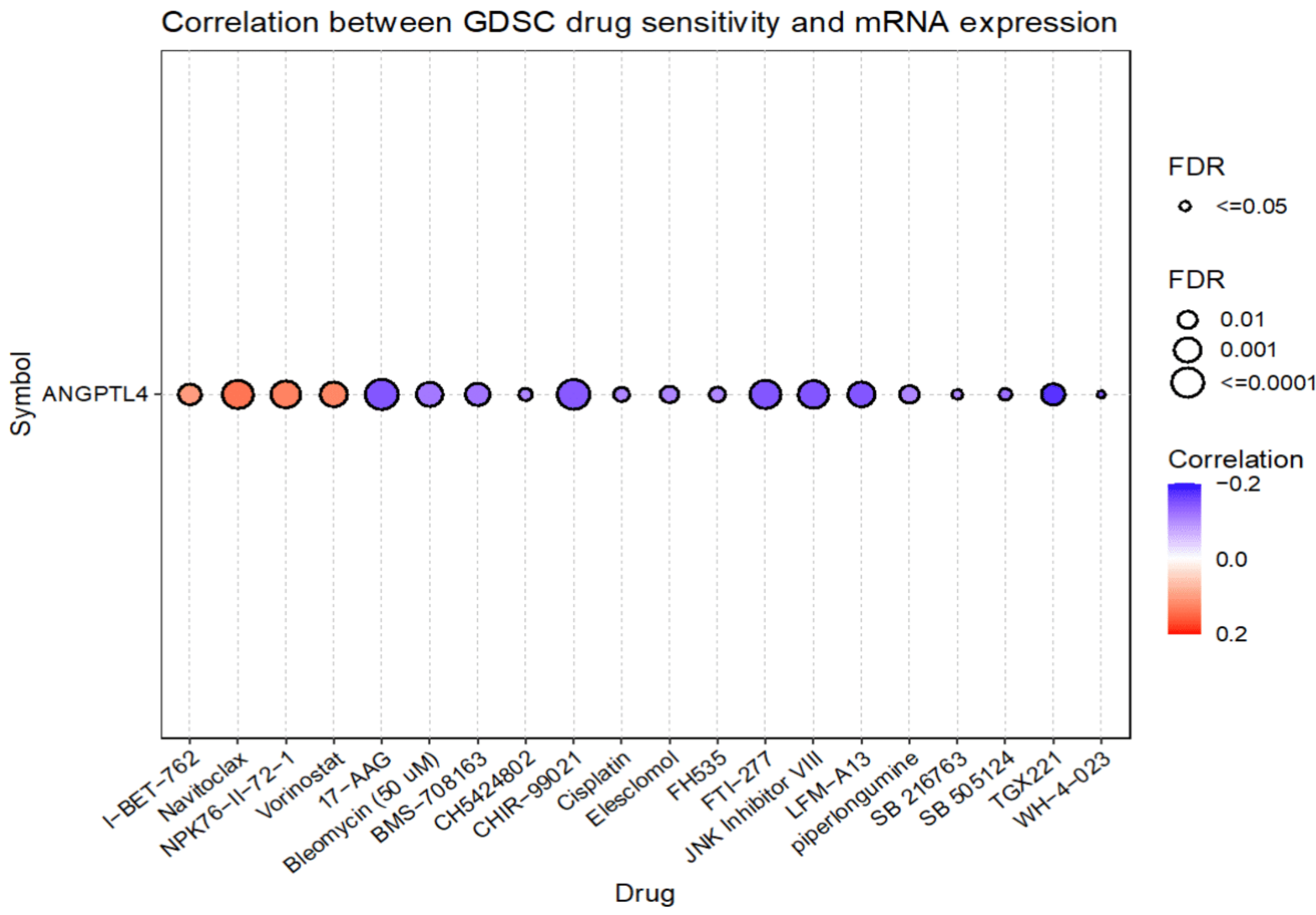

b

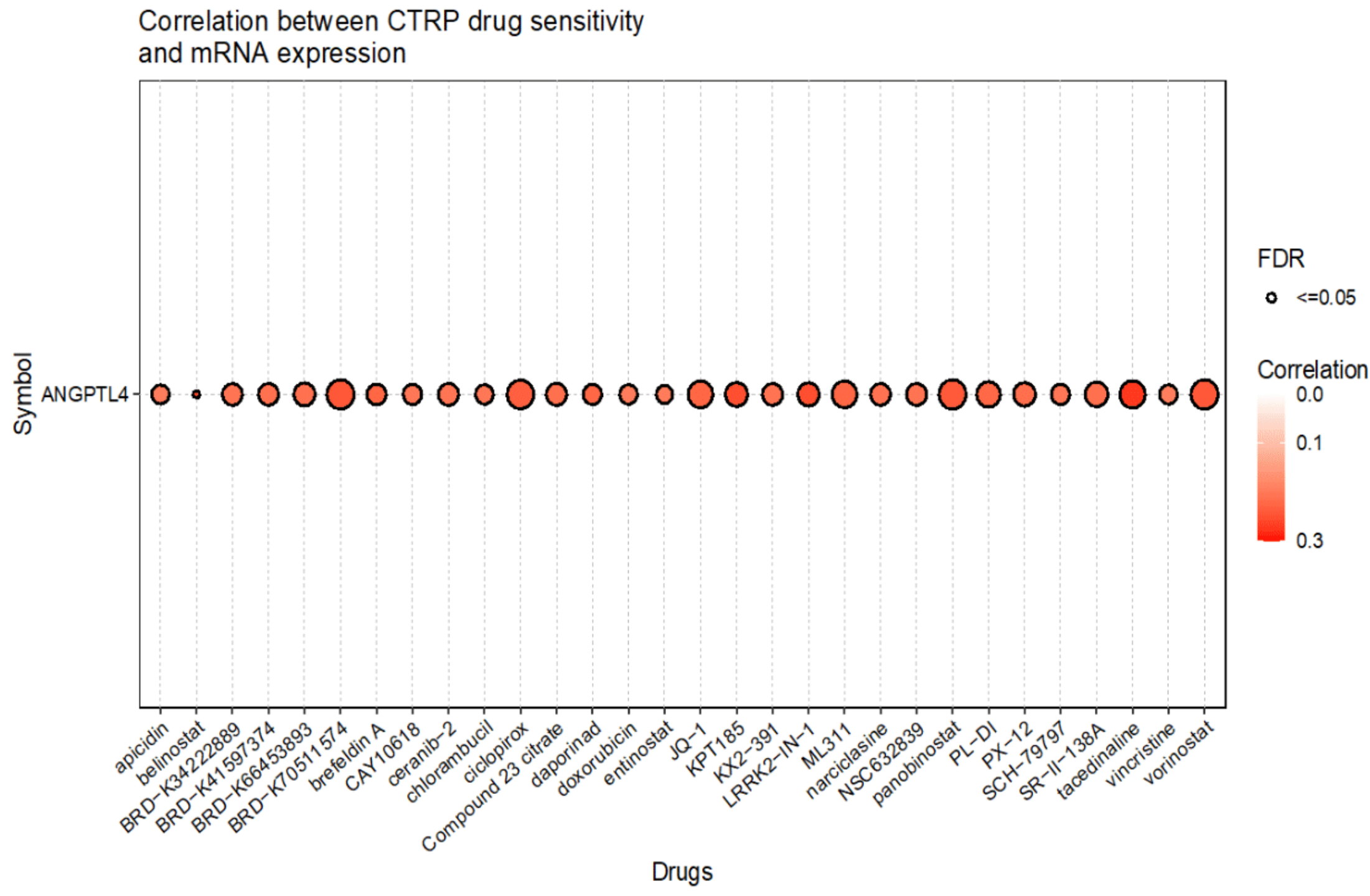

Supplement: S3 Fig — Figure S3 shows two dot plots that represent the correlation between ANGPTL4 expression and drug sensitivity. A positive correlation indicates that increased ANGPTL4 expression leads to increased drug resistance, and vice versa. (A) Shows the drug resistance profile from the GDSC. (B) Shows the drug resistance profile from the CTRP. (PDF) [file pone.0320343.s003.pdf]
