# Supplementary material for: The role of ANGPTL4 in cancer: A meta-analysis of observational studies and multi-omics investigation
Source: PLoS One. 2025 Apr 15;20(4):e0320343. doi: 10.1371/journal.pone.0320343 (PMC11999138; doi:10.1371/journal.pone.0320343)
Supplement: S1 Table — Characteristics of all studies included in the analysis including survival outcomes and the presence of clinicopathological outcomes [64–87]. (DOCX) [file pone.0320343.s004.docx]

| \| **Table 1: Characteristics of all included studies** \| \| --- \| | | | | | | | | | | | | | | | | |
| --- | --- | --- | --- | --- | --- | --- | --- | --- | --- | --- | --- | --- | --- | --- | --- | --- | --- |
| **Study ID** | Year | Country | Cancer type | Tumor stage | Specimen source | Detection method | Sample size | Sex(M/F) | Age | Study period | Follow up (months) | ANGPTL4 | ANGPTL4 expression | | Survival outcomes | Other outcomes |
|  |  |  |  |  |  |  |  |  |  |  |  |  | High | Low |  |  |
| **ZhuominWang et al ^[64]^** | 2010 | China | oral tongue squamous cell carcinoma | I-IV | Tissue | RT-PCR | 158 | (126/32) | NR | 1998-2001 | 99 | median (4.660) | NR | NR | OS | Clinicopathological parameters |
| **Nakayama et al**[**^[65]^**](https://pubmed.ncbi.nlm.nih.gov/20664963/) | 2010 | Japan | Gastric cancer | 0-IV | Tissue | IHC | 103 | NR | NR | 1998-2007 | 43.36 | >10% | 38 | 65 | OS | Clinicopathological parameters |
| **Shibata et al** [**^[66]^**](https://pubmed.ncbi.nlm.nih.gov/20861003/) | 2010 | Japan | esophageal squamous cell carcinoma | I-IV | Tissue | IHC | 104 | (95/9) | 63.6 | 1995-2003 | 27.73 | >30% | 65 | 39 | OS and DFS | Clinicopathological parameters |
| **Li et al** [**^[67]^**](https://pubmed.ncbi.nlm.nih.gov/21674552/) | 2011 | China | Hepatocellular carcinoma | I-IV | Serum | ELISA | 144 | (109/35) | 52.8 | NR | NR | > 93.5 ng/ml | 80 | 64 | N/R | Clinicopathological parameters |
| **Monica Mannelqvist et al ^[68]^** | 2011 | Norway | Endometrial Cancer | I-IV | Tissue | IHC | 286 | NA | NR | 1981–1990 | 204 | staining index > 2 | NR | NR | OS | NR |
| **Nakayama et al ^[49]^** | 2011 | Japan | colorectal cancer | I-IV | Tissue | IHC | 144 | NR | NR | 2001-2009 | 54.23 | >10% | 95 | 49 | OS and DFS | Clinicopathological parameters |
| **Akishima-Fukasawa Y et al** [**^[69]^**](https://pubmed.ncbi.nlm.nih.gov/22034887/) | 2011 | Japan | colorectal cancer | NR | Tissue | IHC | 111 | (76/35) | 67.86 | 1989-2009 | NR | ≥20% | 46 | 65 | NR | Clinicopathological parameters |
| **Yi et al** [**^[70]^**](https://pubmed.ncbi.nlm.nih.gov/23925665/) | 2013 | China | esophageal squamous cell carcinoma | I-IV | Tissue | IHC | 78 | (50/28) | 62 | 2006-2008 | NR | overall score of 3–6 | 43 | 35 | PFS | Clinicopathological parameters |
| **Ng et al** [**^[71]^**](https://pubmed.ncbi.nlm.nih.gov/25148701/) | 2014 | China | Hepatocellular carcinoma | I-IV | Tissue | qRT-PCR | 110 | (88/22) | NR | 1999- 2007 | NR | NR | 43 | 67 | OS and DFS | Clinicopathological parameters |
| **Shafik et al** [**^[72]^**](https://pubmed.ncbi.nlm.nih.gov/26745120/) | 2015 | Egypt | Breast cancer | NR | Tissue | IHC | 54 | NA | 48.6 | NR | NR | overall score of 3-6 | 42 | 12 | NR | Clinicopathological parameters |
| **Tanaka et al** [**^[73]^**](https://pubmed.ncbi.nlm.nih.gov/25060575/) | 2015 | Japan | Oral squamous cell carcinoma | NR | Tissue | IHC | 41 | (18/23) | 61.4 | NR | NR | immunoreactive score of 3-9 | 20 | 21 | NR | Clinicopathological parameters |
| **Li X et al** [**^[74]^**](https://pubmed.ncbi.nlm.nih.gov/26417691/) | 2015 | China | colorectal cancer | I-IV | Tissue | IHC | 54 | (25/29) | NR | NR | NR | overall score "++/+++" | 41 | 13 | NR | Clinicopathological parameters |
| **Kubo H et al** [**^[75]^**](https://pubmed.ncbi.nlm.nih.gov/26893686/) | 2016 | japan | gastric cancer | IB-IIIC | Tissue | IHC | 170 | (113/57) | 71 | 2000-2008 | NR | >31% | 21 | 149 | DFS | Clinicopathological parameters |
| **Zhu X et al** [**^[76]^**](https://pubmed.ncbi.nlm.nih.gov/27166634/) | 2016 | china | non-small cell lung cancer | I-IV | tissue | qRT-PCR | 61 | (41/20) | NR | 2009-2011 | NR | score of 4-9 | 38 | 23 | OS | Clinicopathological parameters |
| **Huang et al** [**^[77]^**](https://pubmed.ncbi.nlm.nih.gov/27505034/) | 2016 | china | tongue squamous cell carcinoma | I-IV | tissue | IHC | 65 | (41/24) | NR | 1996-2006 | 117 | a score of ≥ 3 | 33 | 32 | OS | Clinicopathological parameters |
| **Hata et al ^[78]^** | 2017 | Japan | prostate cancer | NR | tissue | IHC | 70 | NA | 67.5 | 2009-2012 | 53.5 | 20% - 50% | 58 | 12 | RFS | Clinicopathological parameters |
| **Nie et al ^[13]^** | 2019 | china | cervical cancer | IA2–IIA | tissue | IHC | 160 | NA | 47 | 2009-2011 | 59.3 | a final mark of ≥4 | 95 | 65 | OS and DFS | Clinicopathological parameters |
| **Zhao et al ^[11]^** | 2020 | china | breast cancer | I-IV | tissue | IHC | 205 | Only females | 55 | 2003-2003 | 54 | a final socre of 1-2 | 130 | 75 | OS and DFS | Clinicopathological parameters |
| **Cai et al ^[14]^** | 2020 | china | breast cancer | 0-III | tissue | IHC | 161 | Only females | 49 | 2007-2016 | NR | a score of ≥ 4 | 95 | 66 | OS and DFS | Clinicopathological parameters |
| **Aung et al** [**^[79]^**](https://pubmed.ncbi.nlm.nih.gov/35732325/) | 2022 | Thailand | Cholangiocarcinoma | I-IV | serum | IHC | 90 | (60/30) | 61 | NR | NR | >0.6017 AU | 45 | 45 | NR | Clinicopathological parameters |
| **Kamaludin et al** [**^[80]^**](https://pubmed.ncbi.nlm.nih.gov/35366286/) | 2022 | Malaysia | Breast carcinoma | NR | Tissues | IHC | 75 | Only females | 37.24 | 2007-2017 | NR | a total score of ≥4 | 50 | 25 | NR | Clinicopathological parameters |
| **Tanaka et al ^[41]^** | 2022 | Japan | tongue cancer | I-IV | tissue | IHC | 48 | (27/21) | 61.4 | 2003–2015 | NR | >30% | 15 | 33 | NR | Clinicopathological parameters |
| **Dong et al** [**^[81]^**](https://pubmed.ncbi.nlm.nih.gov/28110976/) | 2017 | China | Renal cell carcinoma | I-IV | Serum | ELISA | 110 | (74/36) | 57.2 | 2010-2014 | 40 | > 28.4 ng/ml | 60 | 30 | OS | Clinicopathological parameters |
| **Lee et al** [**^[82]^**](https://pubmed.ncbi.nlm.nih.gov/29277583/) | 2017 | China | urothelial carcinoma | I-IV | Serum | ELISA | 133 | (93/39) | 70.28 | nr | NR | > medium cir-ANGPTL4 Level | NR | NR | DFS | Clinicopathological parameters |
| **Wang et al ^[10]^** | 2021 | china | Gallbladder cancer | I-V | Tissue | IHC | 85 | (29/56) | NR | 2007-2012 | 15 | staining intensity > 3 | 55 | 40 | OS | Clinicopathological parameters |
| **Kirby et al** [**^[83]^**](https://pubmed.ncbi.nlm.nih.gov/27282075/) | 2016 | USA | Pancreatic cancer | I-IV | Tissue | qRT-PCR | 68 | (33/35) | 68 | 2003-2011 | NR | NR | NR | NR | OS | NR |
| **Dao et al** [**^[84]^**](https://pubmed.ncbi.nlm.nih.gov/32405335/) | 2020 | France | Breast cancer | I-IV | Serum | ELISA | 38 | Only females | 53.6 | 2015-2016 | 42 | > 0.1 ng/mL | nr | Nr | OS | NR |
| **Zheng et al** [**^[85]^**](https://pubmed.ncbi.nlm.nih.gov/34645607/) | 2021 | China | colorectal cancer | I-IV | Tissue | IHC | 67 | (41/26) | NR | 2011-2013 | 60 | a grade of ≥ 1 | 34 | 33 | OS | Clinicopathological parameters |
| **Mizuno et al** [**^[86]^**](https://pubmed.ncbi.nlm.nih.gov/35195748/) | 2022 | Japan | colorectal cancer | I-IV | Tissue | IHC | 84 | NR | NR | 2018-2019 | NR | an Allred score ≥ 6 | 35 | 49 | NR | Clinicopathological parameters |
| **Yan et al** [**^[87]^**](https://pubmed.ncbi.nlm.nih.gov/34311032/) | 2021 | Korea | Pancreatic cancer | I-IV | Tissue | IHC | 140 | NR | NR | NR | NR | NR | 70 | 70 | OS | NR |
| IHC: immunohistochemistry; qRT-PCR: quantitative reverse transcription polymerase chain reaction; ELISA: enzyme linked immunosorbent assay; ANGPTL4: angiopoietin like4; OS: overall survival; DFS: disease free survival | | | | | | | | | | | | | | | | |
