# Supplementary material for: The role of ANGPTL4 in cancer: A meta-analysis of observational studies and multi-omics investigation
Source: PLoS One. 2025 Apr 15;20(4):e0320343. doi: 10.1371/journal.pone.0320343 (PMC11999138; doi:10.1371/journal.pone.0320343)
Supplement: S2 Table — Table S2 shows the New Ottawa Scale based quality assessment for all studies. (DOCX) [file pone.0320343.s005.docx]

| **Table 2: the risk of bias and quality of all included studies** | | | | | | | | | | |
| --- | --- | --- | --- | --- | --- | --- | --- | --- | --- | --- |
| **Study ID** | **Representativeness**  **of the exposed cohort** | **Selection of the non-exposed cohort** | **Ascertainment of exposure** | **Outcome of interest is not present at baseline.** | **Adjustment of age and gender** | **Adjustment of other confounding factors** | **Assessment of outcome**  **1** | **Follow-up long enough**  **1** | **Adequacy of follow-up of cohorts**  **1** | **Total**  **8** |
| **Zhuomin Wang** | 1 | 1 | 1 | 1 | 0 | 1 | 1 | 1 | 1 | 8 |
| **Nakayama** | 1 | 1 | 1 | 1 | 0 | 0 | 1 | 1 | 1 | 7 |
| **Shibata** | 1 | 1 | 1 | 1 | 0 | 1 | 1 | 1 | 1 | 8 |
| **Li** | 1 | 1 | 1 | 1 | 0 | 0 | 1 | 0 | 1 | 6 |
| **Monica Mannelqvist** | 1 | 1 | 1 | 1 | 0 | 0 | 1 | 1 | 1 | 7 |
| **Nakayama** | 1 | 1 | 1 | 1 | 0 | 0 | 1 | 1 | 1 | 7 |
| **Akishima-Fukasawa Y** | 1 | 1 | 1 | 1 | 0 | 1 | 1 | 0 | 1 | 7 |
| **Yi** | 1 | 1 | 1 | 1 | 1 | 1 | 1 | 0 | 1 | 8 |
| **Ng** | 1 | 1 | 1 | 1 | 0 | 1 | 1 | 0 | 1 | 7 |
| **Shafik** | 1 | 1 | 1 | 1 | 0 | 0 | 1 | 0 | 1 | 6 |
| **Tanaka** | 1 | 1 | 1 | 1 | 0 | 0 | 1 | 0 | 1 | 6 |
| **Li X** | 1 | 1 | 1 | 1 | 0 | 0 | 1 | 0 | 1 | 6 |
| **Kubo H** | 1 | 1 | 1 | 1 | 1 | 1 | 1 | 0 | 1 | 8 |
| **Zhu X** | 1 | 1 | 1 | 1 | 0 | 0 | 1 | 0 | 1 | 6 |
| **HUANG** | 1 | 1 | 1 | 1 | 1 | 1 | 1 | 1 | 1 | 9 |
| **Hata** | 1 | 1 | 1 | 1 | 1 | 1 | 1 | 1 | 1 | 9 |
| **Nie** | 1 | 1 | 1 | 1 | 0 | 1 | 1 | 1 | 1 | 8 |
| **Zhao** | 1 | 1 | 1 | 1 | 1 | 1 | 1 | 1 | 1 | 9 |
| **Cai** | 1 | 1 | 1 | 1 | 1 | 1 | 1 | 1 | 1 | 9 |
| **Aung** | 1 | 1 | 1 | 1 | 1 | 1 | 1 | 0 | 1 | 8 |
| **Kamaludin** | 1 | 1 | 1 | 1 | 0 | 0 | 1 | 0 | 1 | 6 |
| **TANAKA** | 1 | 1 | 1 | 1 | 0 | 1 | 1 | 0 | 1 | 7 |
| **Dong** | 1 | 1 | 1 | 1 | 0 | 0 | 1 | 1 | 1 | 7 |
| **Hsieh** | 1 | 1 | 1 | 1 | 0 | 0 | 1 | 0 | 1 | 6 |
| **Wang** | 1 | 1 | 1 | 1 | 1 | 1 | 1 | 1 | 1 | 9 |
| **Kirhy** | 1 | 1 | 1 | 1 | 0 | 0 | 1 | 0 | 1 | 6 |
| **Dao** | 1 | 1 | 1 | 1 | 0 | 0 | 1 | 1 | 1 | 7 |
| **Zhang** | 1 | 1 | 1 | 1 | 0 | 0 | 1 | 1 | 1 | 7 |
| **Mizuno** | 1 | 1 | 1 | 1 | 0 | 0 | 1 | 0 | 1 | 6 |
| **Yan** | 1 | 1 | 1 | 1 | 0 | 0 | 1 | 0 | 1 | 6 |
