# Supplementary material for: The role of ANGPTL4 in cancer: A meta-analysis of observational studies and multi-omics investigation
Source: PLoS One. 2025 Apr 15;20(4):e0320343. doi: 10.1371/journal.pone.0320343 (PMC11999138; doi:10.1371/journal.pone.0320343)
Supplement: S3 Table — Table S3 shows the egger’s results for all the meta-analytic outcomes. (DOCX) [file pone.0320343.s006.docx]

| Variable | Intercept | Pvalue |
| --- | --- | --- |
| Gender | 1.2428 | 0.5106 |
| Lymphatic invasion | -0.4989 | 0.8799 |
| Differentiation | -2.2203 | 0.0083 |
| Age | -1.9963 | 0.3614 |
| LNM | 2.2353 | 0.2189 |
| Local recurrence | 1.3782 | 0.8446 |
| Mets | 2.3821 | 0.3863 |
| TNM | 2.7266 | 0.2397 |
| T size | 3.852 | 0.4061 |
| T stage | -0.1195 | 0.9589 |
| Vascular Invasion | -0.7747 | 0.8168 |

Table S3 shows the egger’s test results.
